# Interventions to prevent, delay or reverse frailty in older people: a journey towards clinical guidelines

**DOI:** 10.1186/s12916-019-1434-2

**Published:** 2019-10-29

**Authors:** Maura Marcucci, Sarah Damanti, Federico Germini, Joao Apostolo, Elzbieta Bobrowicz-Campos, Holly Gwyther, Carol Holland, Donata Kurpas, Maria Bujnowska-Fedak, Katarzyna Szwamel, Silvina Santana, Alessandro Nobili, Barbara D’Avanzo, Antonio Cano

**Affiliations:** 1 Department of Health Research Methods, Evidence, and Impact, McMaster University, 1280 Main Street West, Hamilton, ON L8S 4K1 Canada; 2 Geriatric Unit, Fondazione IRCCS Ca’ Granda Ospedale Maggiore Policlinico, Milan, Italy; 3 Nutritional Sciences Doctorate, Università degli Studi di Milano, Milan, Italy; 4 Department of Health Sciences, Università degli Studi di Milano, Milan, Italy; 5 Health Sciences Research Unit: Nursing, Nursing School of Coimbra, Portugal Centre for Evidence Based Practice – a Joanna Briggs Institute Centre of Excellence, Coimbra, Portugal; 6 The Centre for Ageing Research, Lancaster University, Lancaster, UK; 7 Family Medicine Department, Wroclaw Medical University, Wroclaw, Poland; 8 Faculty of Medical Science, Opole Medical School, Opole, Poland; 9 Department of Economics, Management and Industrial Engineering, University of Aveiro, Aveiro, Portugal; 10 Laboratory of Quality Assessment of Geriatric Therapies and Services, Istituto di Ricerche Farmacologiche Mario Negri IRCCS, Milan, Italy; 11 Department of Pediatrics, Obstetrics and Gynecology, University of Valencia, Valencia, Spain

**Keywords:** Frailty, Older people, Interventions, Guidelines, Decision-making, Implementation, GRADE system

## Abstract

**Background:**

Age-related frailty is a multidimensional dynamic condition associated with adverse patient outcomes and high costs for health systems. Several interventions have been proposed to tackle frailty. This correspondence article describes the journey through the development of evidence- and consensus-based guidelines on interventions aimed at preventing, delaying or reversing frailty in the context of the FOCUS (Frailty Management Optimisation through EIP-AHA Commitments and Utilisation of Stakeholders Input) project (664367-FOCUS-HP-PJ-2014). The rationale, framework, processes and content of the guidelines are described.

**Main text:**

The guidelines were framed into four questions – one general and three on specific groups of interventions – all including frailty as the primary outcome of interest. Quantitative and qualitative studies and reviews conducted in the context of the FOCUS project represented the evidence base. We followed the GRADE Evidence-to-Decision frameworks based on assessment of whether the problem is a priority, the magnitude of the desirable and undesirable effects, the certainty of the evidence, stakeholders’ values, the balance between desirable and undesirable effects, the resource use, and other factors like acceptability and feasibility. Experts in the FOCUS consortium acted as panellists in the consensus process. Overall, we eventually recommended interventions intended to affect frailty as well as its course and related outcomes. Specifically, we recommended (1) physical activity programmes or nutritional interventions or a combination of both; (2) interventions based on tailored care and/or geriatric evaluation and management; and (3) interventions based on cognitive training (alone or in combination with exercise and nutritional supplementation). The panel did not support interventions based on hormone treatments or problem-solving therapy. However, all our recommendations were weak (provisional) due to the limited available evidence and based on heterogeneous studies of limited quality. Furthermore, they are conditional to the consideration of participant-, organisational- and contextual/cultural-related facilitators or barriers. There is insufficient evidence in favour of or against other types of interventions.

**Conclusions:**

We provided guidelines based on quantitative and qualitative evidence, adopting methodological standards, and integrating relevant stakeholders’ inputs and perspectives. We identified the need for further studies of a higher methodological quality to explore interventions with the potential to affect frailty.

## Background

### Frailty and its impact

The term ‘frailty’ has been used to describe an age-related state of decline and vulnerability characterised by weakness and decreased physiological reserve [1]. Frail older adults are less able to adapt to stressors such as acute illness, surgery, trauma or bereavement, and are at increased risk of falls, institutionalisation, disability and death [2, 3]. The concept of frailty has been operationalised through different definitions to help clinicians, researchers and other stakeholders to identify frail older patients. One of the most commonly adopted definitions is the one used in the Cardiovascular Health Study, known as the Frailty Phenotype or Physical Frailty. It is based on five criteria related to reduced physical reserve, and defines frail and prefrail states based on the number of criteria met [1]. Others sustain a different approach, which views frailty as a multidimensional risk state defined by the accumulation of deficits in different domains, such as cognition and mood, chronic diseases, polypharmacy, functional autonomy and social conditions, using a continuous scale as opposed to category assignment. The Frailty Index by Rockwood et al. [4] is taken as a paradigm of this second approach.

Although the use of different definitions leads to the identification of different target populations and leads to a variation in the estimate of the actual prevalence of frailty [5, 6], the associations between frailty, adverse outcomes, and health and social care utilisation have been invariably demonstrated. This has been common understanding for more than a decade [7] and has led to several initiatives to tackle frailty and its related outcomes.

### The FOCUS project

To add to the international efforts to reduce the impact of frailty, we conducted the FOCUS (Frailty Management Optimisation through EIP-AHA Commitments and Utilisation of Stakeholders Input) project, funded by the European Union’s Third Health Programme (2014–2020) [8, 9]. The project included a series of quantitative and qualitative studies [10–16] (Table 1), which, through the review and appraisal of the literature and the collection of stakeholder inputs, served the project mission of providing instruments to guide the implementation and scaling up of effective strategies of frailty prevention and management. The development of clinical guidelines represented an ultimate important achievement in the fulfilment of this mission.

**Table 1:** The FOCUS evidence base and its relevance in the assessment of interventions targeting frailty based on the Evidence-to-Decision (EtD) criteria

FOCUS project deliverables^a^	Evidence type	EtD criteria for which the evidence was considered relevant
Systematic review of the effectiveness of interventions on frailty (D4.1.2) [11]	Quantitative: A systematic review of randomised studies on interventions on frailty in older people, including health economy studies	Benefits, harms, resource use, cost-effectiveness, equity
Review of qualitative studies on frailty interventions with stakeholders (D4.1.3) [12]	Qualitative: A meta-synthesis of qualitative studies on stakeholders’ views and experiences of care and interventions in the context of frailty	Outcome importance, value, equity, acceptability, feasibility
Thematic summary of focus groups with stakeholders in three different EU states (D4.1.4) [13]	Qualitative: An inductive thematic analysis of semi-structured focus groups and individual interviews in three European countries (Italy, Poland, UK) with five groups of stakeholders, including frail and non-frail older adults, family caregivers, and health and social care professionals	Outcome importance, value, equity, acceptability, feasibility
Thematic summary of joint focus groups with EU policy-makers (D4.1.5) [14]	Qualitative: An inductive thematic analysis of semi-structured interviews with seven healthcare policy-makers across Europe	Outcome importance, value, equity, acceptability, feasibility
Structured survey of partners within the EIP-AHA (D4.1.7) [15]	Mixed: A structured survey seeking the opinions of EIP-AHA partners	Outcome importance, value, equity, acceptability, feasibility
Realist review (D4.1.8) [16]	Mixed: A realist review combining findings from the different components to examine what works, for whom and in what circumstances	Outcome importance, value, equity, acceptability, feasibility
Comprehensive report of the results of the comparative analyses and modelling (FOCUS internal deliverable D5.2.1–5)	Quantitative: Comparative analyses of EIP-AHA commitments upon structure, process and outcome indicators; modelling analyses of significant predictors of outcome, health and social care needs and use in the frame of frailty, with projected impact of changes in frailty as a result of interventions	Resource use and feasibility (additional considerations)

*EIP-AHA* European Innovation Partnership on Active and Healthy Aging, *EU* European Union

^a^Deliverables are enumerated and titled based on FOCUS Grant Agreement

### The need for clinical guidelines

At the time of designing our project, we recognised the need to offer patients, their formal and informal carers, healthcare practitioners, and decision-makers a scientific support to their actions, by bringing together scientific evidence and stakeholder perspectives into recommendations that responded to the definition of clinical guidelines [17, 18]. In fact, as the interest in the concept of frailty had also grown outside the internal and geriatric medicine field, we had seen several non-geriatric scientific societies including special considerations on the management of the disease(s) of interest related to older frail patients in their clinical guidelines [19–22]. However, within the specific field of frailty as a syndrome, when the FOCUS consortium started its work in 2015, there were no clinical guidelines generated from rigorous methods recognised as standards by the scientific community, which would give frailty the dignity of other clinical conditions [23]. Therefore, we translated our FOCUS project mission into the development of evidence- and consensus-based guidelines, intended for all those involved in decision-making and implementation of actions on frailty.

This correspondence article presents the development process of our FOCUS guidelines, providing their justification, with emphasis on their novelty, limitations and implications.

## Main text

### Definition of the clinical questions: frailty as an outcome

We started by formulating the questions to be answered by the guidelines, based on the PICO format [24] (Table 2). While the project covered the entire spectrum of relevant clinical questions on frailty, including screening and diagnostic strategies [10], we decided to focus the guidelines on the role of interventions to prevent, delay or revert frailty. We framed the guidelines according to a hierarchical structure. We included a first question (General Question, GQ) asking whether the overall current evidence and stakeholder inputs support interventions meant to affect frailty and its course, followed by the addition of sub-questions (Questions 1–3 (Q1–Q3)) focused on specific types of interventions. For each question, two population subgroups were also considered, i.e. frail and prefrail subjects. The choice of the outcome and its definition represented the most sensitive step in the formulation of our PICO questions. We prespecified ‘frailty’ as our primary outcome, in compliance with the FOCUS project mission and the intention to treat frailty as a syndrome; we reflected the choice previously made in the definition of the study inclusion criteria for our systematic review of randomised controlled trials on interventions to prevent or reduce frailty [11]. Specifically, in our systematic review, we included studies that looked at the effect of interventions on frailty defined according to any validated scale, index, indicator or sets of indicators that were explicitly adopted by the authors as a definition for frailty [11]. However, our systematic review and guidelines also considered and evaluated the effect on frailty-related outcomes, which we defined as our secondary outcomes, including cognitive performance, ability to perform activities of daily life and quality of life, among others [11].

**Table 2:** FOCUS guideline questions in the PICO format

GQ – Should interventions to prevent or delay the progression of frailty, or to reverse frailty, be adopted in prefrail or frail older people?	
Q1 – Should physical interventions be recommended to prevent or delay the progression of frailty, or to reverse frailty, in prefrail or frail older people?	
Q2 – Should interventions based on tailored care and/or GEM be recommended to prevent or delay the progression of frailty, or to reverse frailty, in prefrail or frail older people?	
Q3 – Should other interventions be recommended to prevent or delay the progression of frailty, or to reverse frailty, in prefrail or frail older people?	
Patients: People aged 65 years or older, defined as prefrail or frail according to a pre-specified scale, index or criteria, not at the end-of-life phase or selected because of an index disease Interventions: GQ: Any intervention explicitly defined as an intervention for frailty (regardless of the definition of frailty used) Q1: Physical interventions • Interventions based on exercise/physical activity • Nutritional interventions (e.g. diet change, supplementation) • Exercise/physical activity combined with nutritional interventions Q2: Interventions based on tailored care and/or GEM • Uni-professional interventions based on tailored care/GEM • Multidisciplinary interventions based on tailored care/GEM Q3: Other interventions^a^ • Cognitive training • A composite of exercise + nutritional supplementation + cognitive training • Exercise + nutritional consultation • Problem-solving therapy • Hormone therapy • Others Reference intervention: No intervention or placebo or usual care Outcomes: • Frailty – defined according to a composite index, or based on physical performance tests commonly related to the ‘frailty’ definition (SPPB, TUG, gait speed, handgrip strength) • Other relevant patient important outcomes – cognitive performance, functional performance, other measures of physical performance, quality of life, depression, self-perceived health, social engagement, caregiver burden, falls and fractures, mortality, hospitalisation, institutionalisation, comorbidity burden, drug prescription Setting: Any (community, primary care, nursing homes, hospitals) Perspective: Population	

*GEM* geriatric evaluation and management, *GQ* general question, *Q* question, *SPPB* short physical performance battery, *TUG* time up and go

^a^The provided list of interventions includes interventions evaluated in studies found in our systematic review [12] that did not match Q1 and Q2 definitions; it does not include any other possible intervention

### Selection of a framework: GRADE and Evidence-to-Decision

We adopted the GRADE (Grading of Recommendations Assessment, Development and Evaluation) Working Group system to assess the certainty of evidence and define the strength of related recommendations [25, 26]. In particular, we followed the Evidence-to-Decision (EtD) framework, which was developed by the GRADE Working Group to support the process of moving from evidence to decisions, in the contexts of making clinical recommendations or coverage, health system, or public health decisions [27]. It has been implemented as an interactive online tool (iEtD) that can support a consensus-based process, which we used [28]. The framework suggests a list of criteria upon which the intervention(s) of interest should be assessed based on research evidence, namely whether the problem is a priority, the magnitude of the desirable and undesirable effects, the certainty of the evidence, how patients (or others affected, such as carers) value the main outcomes, the balance between desirable and undesirable effects, resource use, acceptability, and feasibility [27].

### FOCUS evidence base

The development of our guidelines was preceded by a series of quantitative and qualitative studies performed within the FOCUS project, reported in separate publications [11–16], which represented the evidence base for our judgments and recommendations. In particular, we performed a systematic review of randomised controlled trials evaluating interventions on frailty, which represented our quantitative evidence base [11]. As a support to the consensus process, the relevant results of the systematic review were synthesised into tables, one for each of the sub-questions (Additional file 1). The tables focused on the effect of interventions on frailty as an outcome, according to different frailty definitions and based on the typical GRADE Summary of Findings table structure [25]. The effect of interventions on the secondary outcomes was narratively synthesised (Additional file 2). We also performed a realist review [16], using the approach of the Realist and Meta-narrative Evidence Synthesis: Evolving Standards (RAMESES) project [29] and integrating the results of the systematic review with other FOCUS studies, to try to respond to the question “what works, for whom and in what circumstances’; this realist review represented the base for our recommendations on implementation (i.e. the conditions for the intervention to succeed). These and the other FOCUS studies are listed in Table 1, together with their relevance to the EtD criteria used to develop these guidelines.

### Consensus process

**Fig. 1:**
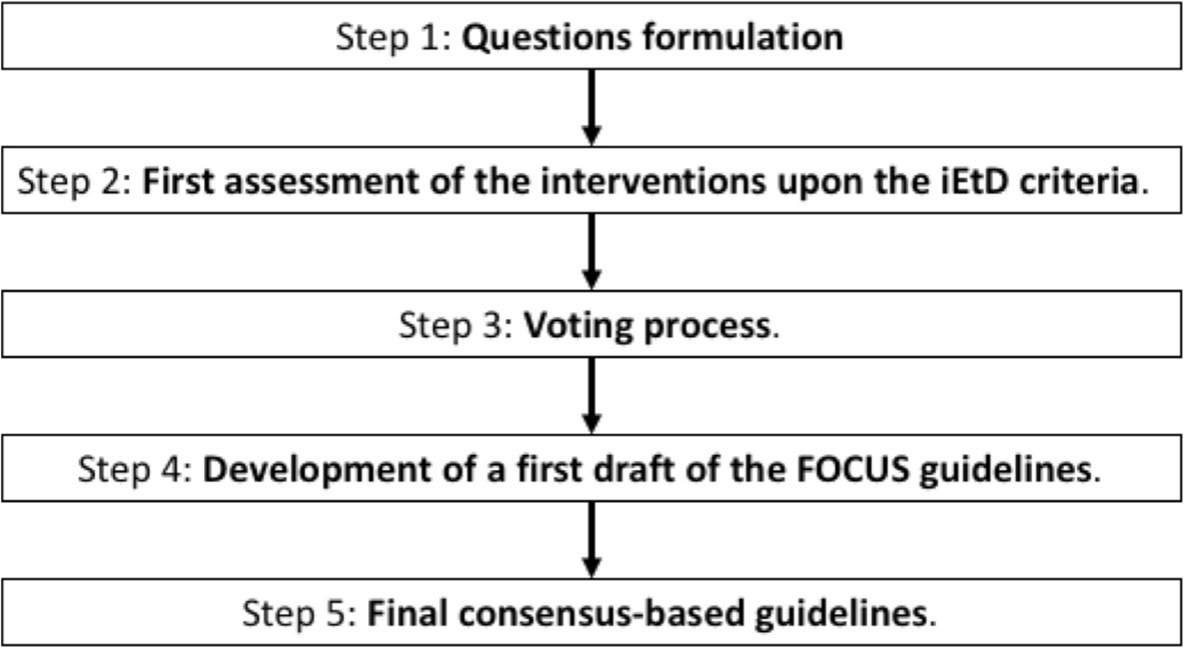
Steps of the FOCUS guideline development process

The guideline panel included the FOCUS project investigators with a background in health and social sciences as well as expertise in frailty, aging and health research methods. Most of them are members of the European Innovation Partnership on Active and Healthy Ageing (EIP-AHA) [30]. Table S1 in the Additional file 3: Table S1 lists the FOCUS investigators, their professional profile and their role in guideline development. We undertook a multistep approach, as schematised in Fig. 1. The technical team (Table S1) conducted a preliminary appraisal of the evidence base upon the iEtD criteria and submitted it to the panel using the iEtD tool. The panel was asked to revise this appraisal and to make their judgment on the existing evidence for each question, upon each criterion. In particular, the judgement was guided by criterion-specific questions (e.g. ‘How substantial are the desirable anticipated effects?’) that the panel had to answer using an ordinal scale. Voting was anonymous. Panel discussion was also encouraged via email. The technical team collated the panel’s judgments and comments. A draft consensus judgement for each criterion and for each question was determined based on the mode and median rating. Any non-coincidence between mode and median rating was considered semi-quantitative proof of skewness of the votes, suggesting the need for discussion with the panel. Even when mode and median coincided, a note (e.g. ‘+’ or ‘–’) was added to the draft consensus statement in the case of significant heterogeneity in the vote distribution.

Of the 13 FOCUS project investigators who were given the role of voting panellists, 11 participated in the voting process. All criteria for all questions were covered by the voting, but with a few missing votes, which were evenly distributed across criteria and questions. We provide an example of the voting outcome, based on the iEtD output and with the addition of the panel’s comments and technical team’s annotations (Additional file 4). The level of agreement was generally very good, but varied slightly across questions and criteria; it was higher for GQ (the highest) and Q1 compared with Q2 and Q3. Although initially all the EtD criteria were judged relevant for guideline development, the FOCUS panellists considered the existing evidence upon the criteria of resource use and cost-effectiveness, as collected in the frame of the FOCUS project, insufficient to use these criteria to justify their recommendations (Additional file 5). Subsequently, the main point raised by the panel during the discussion was the necessity to put more emphasis on what had been learned from the qualitative evidence base, and in particular from our realist review, which combined qualitative and quantitative evidence [16]. This translated into a final version in which the suggestions on how to implement interventions to increase the chance of success were included in the core of the guidelines, rather than only as additional considerations. Finally, the attribute of ‘conditional’ for recommendations, as opposed to ‘strong’, was preferred over ‘weak’ to emphasise that the recommendation is conditional to confirmation from further evidence and/or conditional to implementing facilitators or overcoming barriers.

### FOCUS guidelines and their justification

Table 3 presents the final FOCUS Recommendations (R) on interventions to prevent or delay the progression of frailty, or to reverse frailty, in response to the four questions, i.e. RG (‘G’ for general), R1, R2, and R3. Below, we offer a justification, subgroup considerations and other considerations related to monitoring and evaluation, for each guideline. In Additional file 5 we provide a detailed justification for the judgement on each criterion for each guideline.

**Table 3:** FOCUS guidelines

**GQ – Should interventions to prevent or delay the progression of frailty, or to reverse frailty, be adopted in prefrail or frail older people?**	
**Recommendation RG**	**Conditions for the intervention to succeed** [14]
We suggest implementing interventions specifically intended to have an impact on frailty in older age, i.e. preventing or delaying the progression of frailty, or reversing frailty (conditional strength of recommendation)	**Participant factors** • Co-create the details of interventions with intended recipient groups • Consider the level of frailty of participants • Employ best practice health psychology behaviour change strategies • Consider group and fun, rewarding interventions wherever possible • Determine whether the intervention is only appropriate if a specific deficiency is present, e.g. nutritional deficiency • Before implementing an intervention, consider correcting deficiencies that can interact with the intervention’s working mechanisms • Address understanding and attitudes towards malleability of frailty of patients before the intervention • Address self-efficacy in the intervention activity • Consider accessibility of locations to individuals • Employ approaches that are person, family and ‘lifeworld’ centred • Include social and psychological wellbeing factors **Organisational factors** • Co-create the details of interventions with intended delivery practitioners • Ensure training to emphasise implementation fidelity and standardisation of delivery • Address practitioners’ understanding and attitudes towards the malleability of frailty before the intervention • Consider expectations of older adults’ commitment and ability to participate • Provide some training in health psychology components **Country/contextual/cultural factors** • Consider the suitability of the intervention for your context (e.g. clinical, community, inpatient, outpatient) • Consider likely cultural preferences, e.g. for expert-led or self-directed interventions • Consider what ‘normal care’ is in your context • Consider the level of existing health literacy in your population
**Q1 – Should physical interventions be recommended to prevent or delay the progression of frailty, or to reverse frailty, in prefrail or frail older people?**	
**Recommendation R1**	**Conditions for the intervention to succeed** [14]
We suggest implementing physical interventions, including physical activity/exercise, nutritional interventions, and a combination of exercise and nutritional interventions, to prevent or delay the progression of frailty, or to reverse frailty (conditional strength of recommendation). The recommendation is stronger for group-based supervised exercise programmes, either alone or in association with nutritional supplementation	Among the factors to consider when an intervention on frailty is implemented, those which are particularly relevant in the case of physical interventions are: • Consider implementing those factors that can increase participants’ acceptance of and, as a consequence, compliance to the intervention, i.e. the inclusion of elements favouring or promoting socialisation, fun, accessibility, self-efficacy and commitment. Among those factors, consider the implementation of group-based exercise programmes and supervision by professionals with adequate training; in general, include professionals with adequate skills in health psychology and communication; the inclusion of these elements might affect the adherence to the intervention • Consider the characteristics of the participants to whom the intervention will be directed, for example, the presence of deficits that make the intervention necessary, and the level of expected compliance. The level of frailty particularly may have an important impact, not least because it is likely to be associated with these other characteristics • Even though physical interventions for physical components of frailty are appropriate, considering the person as a whole, including, for example, their psychological wellbeing and functions and their social context, may be beneficial • The opportunity to take into account and include these elements might affect the feasibility and suitability of the interventions in each specific context
**Q2 – Should interventions based on tailored care and/or GEM be recommended to prevent or delay the progression of frailty, or to reverse frailty, in prefrail or frail older people?**	
**Recommendation R2**	**Conditions for the intervention to succeed** [14]
We suggest implementing interventions based on tailored care and/or GEM, to prevent or delay the progression of frailty, or to reverse frailty (conditional strength of recommendation). The recommendation is stronger for GEM-based interventions involving a multidisciplinary team, especially in inpatient clinical settings, but still conditional to the confirmation from further studies of good quality	Among the factors to consider when an intervention on frailty is implemented, those of particular relevance to interventions aimed at a more comprehensive concept of frailty: • Consider the context (the clinical setting, usual care, cultural preferences) • Co-create the details of interventions with intended delivery practitioners, provide adequate training to ensure implementation fidelity • Consider the inclusion of professionals with adequate skills or provide training in health psychology and communication • Employ approaches that are person, family and ‘lifeworld’ centred
**Q3 – Should other interventions be recommended to prevent or delay the progression of frailty, or to reverse frailty, in prefrail or frail older people?**	
**Recommendation R3**	**Conditions for the intervention to succeed** [14]
We suggest considering interventions to prevent or delay the progression of frailty, or to revert frailty, based on cognitive training, alone or in combination with exercise and nutritional supplementation (conditional strength of recommendation), and on exercise combined with diet consultation, at least in prefrail populations. At the moment, the panel does not suggest adopting interventions based on hormone therapy or on problem-solving therapy with the aim of preventing or delaying the progression of frailty or of reverting frailty (conditional strength of recommendation). Currently, there is no evidence in favour or against other interventions potentially effective on frailty (e.g. other types of psychological interventions, interventions mainly focused on increasing socialisation, other types of hormone therapies and pharmacological interventions).	Many of the factors described for the success of a physical intervention (refer to Q1), or for interventions based on a patient-centred approach and comprehensive care (refer to Q2), are also relevant to alternative interventions described in Q3.

*GQ* general question; *Q* question; *R* recommendation

**RG – We suggest implementing interventions specifically intended to have an impact on frailty in older age, i.e. preventing or delaying the progression of frailty or reversing frailty (conditional strength of recommendation).**

#### Justification.

The recommendation stems from the belief, common among different stakeholders and endorsed by the guideline panel, that frailty as an outcome is important and that interventions explicitly conceived to address it are needed. We found some quantitative evidence for different interventions being associated with delaying the progression of frailty or reverting frailty, although the effects were moderate in their size at maximum. These interventions, based on either a mainly physical concept of frailty or on a more comprehensive view, could be potentially fair, acceptable and feasible from a population perspective. However, the strength of this recommendation is weak/conditional because of the low certainty/quality of the quantitative evidence, due to substantial study limitations, inconsistency and heterogeneity across studies (different populations, interventions, outcome definition, and settings/contexts) as well as imprecision of the estimates.

#### Subgroup considerations.

The level or extent of frailty was expected to act as an effect modifier of interventions on frailty, i.e. affecting their relative effect, because of several reasons. For instance, some interventions are expected to work only or more in the presence of a certain deficit status. On the other hand, being frailer might affect the opportunity to benefit from interventions that either require an active participation or work only in less severe illness states. In fact, among the studies examined in our systematic review [11], those that found significant benefits on frailty progression included participants belonging to a range of levels of frailty and ages. It was difficult to look at the effect of the level of frailty as a predictor of an intervention to succeed since the heterogeneity across studies upon several aspects meant that the role of the level of frailty could have been confounded by other possible predictors such as the level of compliance or adherence to the intervention. Moreover, even if some of the authors themselves supposed or perceived there to be a window for the intervention to work according to the level of frailty or disability of participants, this hypothesis was not systematically explored in any study.

#### Considerations on monitoring and evaluation.

The panel judged the implementation of strategies to monitor (and then improve) the adherence to the intervention as important, in particular in the case of interventions that require an active participation of the older person. The need to implement an effective evaluation plan of the interventions was also emphasised. In the FOCUS project, we aimed to analyse the initiatives (i.e. commitments or Good Practices) within the EIP-AHA [30] in order to study the relationship between the outcomes of these initiative and feasibility aspects (barriers and facilitators), resources and processes (Table 1). In this context, we found that many initiatives were undertaken without any plan for evaluation, e.g. assessment of their outcomes in the long run, outside of a research context, and/or compared with the resources used. This limits the opportunity to assess the transferability and scalability of interventions, which is important from a population perspective, and is one of the main objectives of the EIP-AHA initiative of the European Commission.

**R1 – We suggest implementing physical interventions, including physical activity/exercise, nutritional interventions, and a combination of exercise and nutritional interventions, to prevent or delay the progression of frailty or to reverse frailty (conditional strength of recommendation). The recommendation is stronger for group-based supervised exercise programmes, either alone or in association with nutritional supplementation.**

#### Justification.

We found quantitative evidence supporting the success of physical interventions in delaying the progression of or revert frailty, especially when frailty was defined according to a physical paradigm or measures of its physical component. This component, according to older people’s opinions collected through our qualitative studies (Table 1), although partial, appears to have a compensatory and synergistic relationship with other frailty components such as psychological frailty. However, the strength of this recommendation is weak because of the low certainty/quality of the quantitative evidence, due to substantial study limitations, inconsistency and heterogeneity (different populations and interventions) as well as imprecision. Furthermore, the recommendation is meant to be conditional to the presence of factors shown to affect the acceptability and feasibility of such interventions and, hence, their success (Table 3).

#### Subgroup considerations.

Among the studies on physical interventions included in our systematic review [11], those that found significant benefits for frailty progression included participants belonging to a range of severities of frailty and ages. This was true for the different types of physical interventions considered here. On the other hand, it was suggested that a deficit status was necessary for interventions based on nutritional supplementation to effectively impact the level of frailty [31]. Even if some of the authors themselves supposed or perceived there to be an intervention window for physical transitions, this hypothesis was not systematically explored in any study. Additionally, without any specific analysis, it was impossible to separate the role of the severity of frailty on the effectiveness of the intervention from other possible correlated predictors. For example, in one study on exercise plus protein supplementation it was clearly shown that the initial level of frailty was associated with the level of compliance to the intervention [32].

**R2 – We suggest implementing interventions based on tailored care and/or Geriatric Evaluation and Management (GEM), to prevent or delay the progression of frailty or to reverse frailty (conditional strength of recommendation). The recommendation is stronger for GEM-based interventions involving a multidisciplinary team, especially in inpatient clinical settings, but still conditional to the confirmation from further studies of good quality.**

#### Justification.

We found some quantitative evidence for interventions based on a tailored care concept and/or on GEM being able to delay the progression of or revert frailty, as defined according to either a mainly physical paradigm or a multidomain paradigm. Some studies also reported on the impact of these interventions on frailty-related outcomes like functional ability, quality of life and hospitalisation (Additional file 2). Involvement of different professionals, a patient-centred approach, and delivering the intervention at a convenient location and/or in inpatient settings, seemed to be factors more likely associated with successful interventions, even though only limited head-to-head comparisons between interventions differing upon these aspects exist. Such types of interventions and, in particular, the presence of those factors, are also consistent with older people’s values and preferences as well as policy-makers’ idea of the direction towards which health systems should go, as collected through qualitative research [12–14] (Table 1). However, quantitative findings were not consistent across studies, even across similar studies, and studies had several methodological pitfalls. Therefore, no strong recommendations could be made.

#### Subgroup considerations.

It was difficult to reliably prove whether the level of frailty really acted as an effect modifier since, within studies, no subgroup analyses were systematically undertaken with this purpose. As a matter of fact, most of the interventions of this category that had positive significant effects on frailty were implemented in frail rather than prefrail populations.

**R3 – We suggest considering interventions to prevent or delay the progression of frailty or to revert frailty, based on cognitive training, alone or in combination with exercise and nutritional supplementation (conditional strength of recommendation), and on exercise combined with diet consultation, at least in prefrail populations. At present, the panel does not suggest adopting interventions based on hormone therapy or on problem-solving therapy with the aim of preventing or delaying the progression of frailty or of reverting frailty (conditional strength of recommendation). Currently, there is no evidence in favour or against other interventions that could potentially be effective on frailty (e.g. other types of psychological interventions, interventions mainly focused on increasing socialisation, other types of hormone therapies, and pharmacological interventions).**

#### Justification.

Interventions included in this last question were assessed separately because of their heterogeneity. Only one study per intervention was available. The panel judgment was mainly based on the quantitative results of these single studies and their quality, along with considerations regarding values, equity, acceptability and feasibility that could apply to each intervention separately. Thus, the evidence was judged as uncertain and the recommendations were made with weak strength, conditional to the confirmation from further studies.

#### Subgroup considerations.

All the studies relevant to this question were performed on a population definable as prefrail; therefore, no subgroup considerations could be made.

## Discussion

### Strengths of our work

This paper presents evidence- and consensus-based clinical guidelines on interventions to prevent or treat frailty, developed in the context of the FOCUS project. This was a core objective of the project, i.e. an ultimate deliverable in which the quantitative and qualitative research studies conducted in context of the project converged, and also represented the content of the service we intended to deliver in our proposal. To achieve such a pivotal objective, we required a solid methodological structure and the involvement of the entire FOCUS consortium to act as the guideline panel. We developed four guidelines based on one overarching question concerning interventions for frailty in general and three sub-questions on specific groups of interventions. Each guideline includes a recommendation on whether and how interventions should be implemented.

Our work has distinctive characteristics among other initiatives that have been undertaken to help clinicians and patients make informed decisions in the context of frailty. For instance, in 2015, the EIP-AHA – Action Group A3 produced a Decalogue on Frailty Prevention as a result of the work conducted by the group in the period 2012–2015, which includes ten key messages focusing on “*the main areas of interest that policy makers at Member State level would need to support in order to tackle frailty*” [33]. Before that, the British Geriatrics Society, in association with the Royal College of General Practitioners and Age UK, had published Fit for Frail, a comprehensive best practice guidance document for the care of older people living with frailty in community and outpatient settings [34]. Both of these initiatives are consensus-based guides that stemmed from the expertise and experience of the groups’ or societies’ members. Although these documents are useful and translate knowledge into action, they either did not start from a systematic appraisal of the evidence, or the process and evidence was not clearly documented and accessible. More recently, the results of a concurrent initiative – the Asia-Pacific Clinical Practice Guidelines for the Management of Frailty – have been published [35]. Starting from the presentations and discussions at the Asia-Pacific Geriatrics Conference on Geriatrics Beyond Borders: Are We Frailty Ready?, they performed a comprehensive and systematic review and adopted the GRADE approach to develop recommendations that span many aspects of the management of frailty. Two elements, inherent to the FOCUS mission, distinguish ours from these guidelines. First, we had the opportunity to use both quantitative and qualitative evidence in order to combine effect sizes with relevant stakeholders’ inputs and perspectives in the development of our guidelines, according to an integrated knowledge translation strategy. For these reasons, we adopted the EtD framework proposed by the GRADE working group, i.e. because of the relevance given to perspectives and subgroups, and the value assigned to criteria like values, acceptability, feasibility and equity. Secondly, not only did we have a narrower and deeper focus on interventions only, but we specifically reviewed and based our recommendations on interventional studies that explicitly looked at frailty as an outcome, excluding those studies in which interventions were applied to people with or at risk of frailty but assessed upon different outcomes. Although this may have led to the inclusion of a narrower evidence base, our approach intended to give frailty the dignity of a condition. Indeed, the role of frailty as a measurable outcome, and not only as a predictor, to evaluate the impact of interventions or of other types of exposures, has been increasingly recognised in different clinical settings [36, 37]. At the same time, within the selected studies, we did consider other outcomes, thus not overlooking or undervaluing the possibility that interventions can impact other important adverse events to which frail people are vulnerable.

### Limitations

Our work has recognised limitations. The main limitation to the production of definitive guidelines was related to the quantitative evidence base. The evidence available was of low quality due to methodological bias, inconsistencies and imprecision in the existing studies. In addition to the low quality of the collected evidence, there was a high level of methodological heterogeneity, which had already prevented any quantitative synthesis in our systematic review [11]. The diversity of frailty definitions and operationalisations was mostly expected. It is also possible that our choice to include studies in our systematic review in which authors operationalised frailty using a study-specific, pre-specified set of indicators, while valuing the intention of the investigators, increased such heterogeneity. As a result of the heterogeneity and low quality of the collected evidence, which was often coupled with inadequate reporting, we were not able to draw the typical GRADE summary of findings or evidence profile tables [25]. The heterogeneous nature of interventions also represented a challenge to framing the guidelines into questions and grouping the interventions in an appropriate but also user-friendly manner. Finally, we encountered some technical limitations. Firstly, there were time constraints dictated by the FOCUS project timeline, which impeded the possibility of arranging a conference to facilitate the panel discussion. However, the discussion that took place online and by email achieved satisfactory results. Secondly, although extremely useful and user friendly, the EtD tool was limited in terms of flexibility to the development of guidelines for heterogeneous interventions in a field of complex nosography such as frailty in older age.

### Lessons learned and future directions

The ultimate lesson we learned with our 3-year work is that, while stakeholders showed awareness of and place relevance to the challenge represented by age-related frailty, the current scientific quantitative evidence still has important limitations. This was reflected in our guidelines, which could include only weak or conditional recommendations. Even if such types of recommendations might seem less appealing or helpful, they do yield an important message to the clinician and decision-maker, i.e. to be considered when these interventions are adopted, or indeed, to give consideration to when and whether to adopt them. This message reflects the limitations of the scientific literature and important implementation considerations. Our work also conveys an important lesson to the researcher – while the evidence heterogeneity could never be eliminated since it reflects the heterogeneity in the definition of frailty itself, we need more studies which specifically recognise and measure frailty as an outcome as well as greater adherence to high methodological standards. This is true in general, but in particular for studies on comprehensive frailty interventions, which have the potential to be effective, especially when defined holistically, and have so far not been adequately evaluated. We also need more studies to compare different interventions. The fact that most of the studies included in our evidence base compared the experimental intervention with ‘usual practice’, which can differ dramatically from reality to reality, made the findings extremely context bound. This generated the need to include, in our guidelines, not only an answer to the question ‘does it work?’ but also a consideration of the circumstances of success.

## Conclusion

Herein, we delivered evidence- and consensus-based guidelines on interventions that can affect frailty as an outcome. We recognised that these are not conclusive; rather, they should foster further studies, both on those interventions already investigated in the literature but requiring higher research standards as well as on potentially effective interventions that have not been appropriately studied thus far. On the other hand, our recommendations should start informing practices. In the context of the FOCUS project, we ended our project by testing the feasibility and impact of our guidelines when used to inform clinical decisions and help implement or refine interventions addressing frailty as a patient-important outcome; the results of these proof-of-concept pilots are currently in preparation for publication [38].

## Supplementary information

**Additional file 1.** Synthesis of quantitative evidence from the FOCUS systematic review [9], showing the effect of the interventions on frailty as an outcome, adapted from the typical GRADE Summary of Findings table structure, as offered to the guideline panellists.

**Additional file 2.** Narrative synthesis of the effect of interventions on secondary outcomes, as offered to the guideline panellists.

**Additional file 3: Table S1.** Table that describes the members of the FOCUS Guideline Panel and their role in the development of the guidelines.

**Additional file 4.** Example of the output of the voting process using the Evidence to Decision template, as received and processed by the guidelines technical team.

**Additional file 5.** Detailed justification for the judgement upon each Evidence to Decision criterion, for each guideline.

## Data Availability

Not applicable.

## Abbreviations

EIP-AHA European: European Innovation Partnership on Active and Healthy Ageing,
EtD: Evidence-to-decision
FOCUS: Frailty management optimisation through EIP-AHA commitments and utilisation of stakeholders input
GEM: Geriatric evaluation and management
GRADE: Grading of recommendations assessment, development and evaluation
iEtD: interactive EtD
