# Supplementary material for: Interventions to prevent, delay or reverse frailty in older people: a journey towards clinical guidelines
Source: BMC Med. 2019 Oct 29;17:193. doi: 10.1186/s12916-019-1434-2 (PMC6819620; doi:10.1186/s12916-019-1434-2)
Supplement: Supplementary file 1 — Additional file 1. Synthesis of quantitative evidence from the FOCUS systematic review [9], showing the effect of the interventions on frailty as an outcome, adapted from the typical GRADE Summary of Findings table structure, as offered to the guideline panellists. [file 12916_2019_1434_MOESM1_ESM.pdf]

Q1.1 Should interventions based on phsyical activity/exercise be recommended to prevent or delay the progression of or to revert frailty?

OUTCOME	Study	Intervention	Duration of intervention (total)	Comparison	Setting	Frailty definition	Frail - Prefrail	Pt n (total)	Pt n (intervention)	Pt n (control)	FUP duration	Narrative description of results	Considering the evidence on the impact on Frailty as a whole (regardless of the definition used for the outcome)		
													Quality of evidence (GRADE) - overall	Quality of evidence (GRADE) - Prefrail	Quality of evidence (GRADE) - Frail
**Frailty according to a composite index (continuous)**	[45] Ng	Multicomponent exercise intervention, moderate, gradually increasing intensity, tailored to participants' individual abilities. Resistance exercises, balance training exercises involving functional strength, sensory input, and added attentional demands Group, supervised by a qualified trainer (for 12 weeks) then alone	12 weeks in classes, followed by 12 weeks of home-based exercises.	Usual care	Community dwelling	Fried (CHS)	Prefrail	98	48	50	1 year	M (SD)<br>Intervention<br>Pre 2.2 (0.85)<br>3 m 1.2 (0.75)<br>6 m 1.3 (0.87)<br>12 m 1.4 (0.80)<br>Control<br>Pre 1.8 (0.80)<br>3 m 1.3 (0.85)<br>6 m 1.4 (1.06)<br>12 m 1.6 (0.97)<br>Significant difference in change between intervention and control at 3 m, 6 m and 12 m.			
**Frailty according to a composite index (dichotomous - transition to a less severe frailty category)**	[45] Ng	Multicomponent exercise intervention, moderate, gradually increasing intensity, tailored to participants' individual abilities. Resistance exercises, balance training exercises involving functional strength, sensory input, and added attentional demands Group, supervised by a qualified trainer (for 12 weeks) then alone	12 weeks in classes, followed by 12 weeks of home-based exercises.	Usual care	Community dwelling	Fried (CHS)	Prefrail	98	48	50	1 year	Transition to a lower frailty category<br>interv: 41.3%<br>contr: 15.2%<br>Significant OR<br>4.05 (1.50-10.8)			
	[40] Kim Suzuki	Multicomponent exercise intervention, moderate intensity (warm-up, strengthening exercises, balance and gait training, cool-down).<br>Group, supervised (instructor + trainers), delivered at a research institute of gerontology	3 months	Usual care	Community dwelling	Fried	Frail	66 (63)	33 (31)	33 (32)	3 months (+ 4 months of follow up post intervention)	Reversal post intervention<br>interv: 51.5%<br>contr: 30.3%<br>Odds Ratio (95% CI)<br>2.44 (0.89–6.70)<br>Reversal post 4 m:<br>interv: 39.4%<br>contr: 15.2%<br>OR 3.64 (1.12–11.85)			
**Short Physical Performance Battery**	No study														
	[39] Hars	Multitask exercise program based on Jaques-Dalcroze eurhythmics (walking following the piano music, responding directly or oppositely to changes in music's rhythmic patterns, phrases, form or other aspects)<br>Group, in community centres, supervised by a certified instructor	6 months	Usual care (discontinuation of intervent)	Community dwelling	Fried	Prefrail	101 (52)	26 (23)	75 (29)	4 years	mean ± SD (sec)<br>Intervention<br>Pre 9.7 ± 2.2<br>Post 1 y 9.3 ± 1.9<br>Post 4 y 10.0 ± 2.0<br>Control<br>Pre 10.5 ± 2.1<br>Post 1 y 9.8 ± 1.8<br>Post 4 y 12.7 ± 3.7<br>Significant difference in change bewteen intervention and control, from baseline to 4 years and from 1 y to 4 years (even if from 1 to 4 y the performance worsened in both groups).			

**Time up and Go**	[40] Kim Suzuki	Multicomponent exercise intervention, moderate intensity (warm-up, strengthening exercises, balance and gait training, cool-down).<br>Group, supervised (instructor + trainers), delivered at a research institute of gerontology	3 months	Usual care	Community dwelling	Fried	Frail	66 (63)	33 (31)	33 (32)	3 months (+ 4 months of follow up post intervention)	Mean [sec] ± SD<br>Intervention<br>Pre 9.89 ± 2.27<br>Post 7.87 ± 1.83<br>Post 4 m 7.04 ± 1.45<br>Control<br>Pre 10.44 ± 3.79<br>Post 10.00 ± 4.32<br>Post 4 m 7.99 ± 3.79<br>Significant difference in mean change bewteen pre- and post-intervention in the intervention compared with the control - No effect at FUP			
	[32] Cadore et al., 2014	Multicomponent exercise intervention (high-speed resistance training, balance, and gait exercises, on muscle strength)<br>Individual, at the nursing home, supervised by one experienced physical trainer	12 weeks	Usual care (mobility exercise)	Institutionalized	Fried	Frail	32 (24)	16 (11)	16 (13)	12 weeks	Intervention<br>Pre 19.9±8.0 sec<br>Post 18.8±7.9 sec<br>Control<br>Pre 18.4±5.1 sec<br>Post 21.8±6.3 sec<br>Significant difference in change pre-post between intervention and control			
	[34] Clegg	Strengthening exercises for the muscle groups required for basic mobility skills (exercise manual)<br>Inidvidual, at home, with the support (weekly home visits and telehone calls) of physiotherapists	12 weeks	Usual care	Mixed population (community dewlling people in assisted living conditions)	Edmonton	Frail	84 (70)	45 (40)	39 (30)	12 weeks	Intervention<br>Pre 52.0 (62.4) sec<br>Post 62.4 (77.7) sec<br>Change −10.4 (64.0) sec<br>Control<br>Pre 57.9 (74.1) sec<br>Post 97.0 (116.7) sec<br>Change −39.1 (90.6) sec<br>Diff unadj [mean (95% CI)]<br>28.7 (−8.2, 65.5)<br>Diff adj [mean (95% CI)]<br>28.6 (−8.5, 65.9)			
	[39] Hars	Multitask exercise program based on Jaques-Dalcroze eurhythmics (walking following the piano music, responding directly or oppositely to changes in music's rhythmic patterns, phrases, form or other aspects)<br>Group, in community centres, supervised by a certified instructor	6 months	Usual care (discontinuation of intervent)	Community dwelling	Fried	Prefrail	101 (52)	26 (23)	75 (29)	4 years	mean ± SD (sec)<br>Intervention<br>Pre 9.7 ± 2.2<br>Post 1 y 9.3 ± 1.9<br>Post 4 y 10.0 ± 2.0<br>Control<br>Pre 10.5 ± 2.1<br>Post 1 y 9.8 ± 1.8<br>Post 4 y 12.7 ± 3.7<br>Significant difference in change bewteen intervention and control, from baseline to 4 years and from 1 y to 4 years (even if from 1 to 4 y the performance worsened in both groups).	⨁⨁⊖⊖<br>**LOW**<br>**Due to study limitations, inconsistency (different populations** [illegible]	⨁⨁⊖⊖/⨁⨁⨁⊖<br>**LOW to MODERATE**<br>**Due to study limitations (substantial in all studies except in Ng et al.), inconsistency**	⨁⨁⊖⊖<br>**LOW**<br>**Due to study limitations (substantial in all studies), inconsistency** [illegible]

Walking Speed	[37] Giné-Garriga	Functional balance and strength-based exercises, Group ,in an indoor primary-care facility, supervised (investigator and an assistant certifed in first aid)	12 weeks	Usual care (+ health education)	Community dwelling	poor physical ability and self-reported exhaustion	Frail	51 (41)	25 (22)	25 (19)	12 weeks (last follow up afer 36 weeks)	mean (SD) [sec]<br>Intervention<br>Pre 11.73 (0.60)<br>Post 12 9.20 (0.60)<br>Post 36 10.05 (0.62)<br>Control<br>Pre 11.87 (0.65)<br>Post 12 12.39 (0.65)<br>Post 36 12.76 (0.74)<br>Significant difference in the pre-post changes between intervention and control, both at 12 and 36 months	and interventions), and imprecision	(different interventions), and imprecision	(different populations and interventions), and imprecision
	[45] Ng	Multicomponent exercise intervention, moderate, gradually increasing intensity, tailored to participants' individual abilities. Resistance exercises, balance training exercises involving functional strength, sensory input, and added attentional demands Group, supervised by a qualified trainer (for 12 weeks) then alone	12 weeks in classes, followed by 12 weeks of home-based exercises.	Usual care	Community dwelling	Fried (CHS)	Prefrail	98	48	50	1 year	M (SD) [sec]<br>Intervention<br>Pre 6.1 (2.08)<br>3 m 4.8 (0.89)<br>6 m 5.0 (1.04)<br>12 m 4.9 (0.99)<br>Control<br>Pre 5.6 (2.07)<br>3 m 5.1 (2.09)<br>6 m 4.9 (1.47)<br>12 m 5.2 (1.72)<br>Significant difference in change between intervention and control at 6 m and 12 m.			
	[40] Kim Suzuki	Multicomponent exercise intervention, moderate intensity (warm-up, strengthening exercises, balance and gait training, cool-down).<br>Group, supervised (instructor + trainers), delivered at a research institute of gerontology	3 months	Usual care	Community dwelling	Fried	Frail	66 (63)	33 (31)	33 (32)	3 months (+ 4 months of follow up post intervention)	Intervention<br>Pre 1.17 ± 0.21<br>Post 1.26 ± 0.27<br>Post 4 m 1.21 ± 0.22<br>Control<br>Pre 1.18 ± 0.24<br>Post 1.13 ± 0.22<br>Post 4 m 1.18± 0.23 No significant difference in mean change bewteen pre- and post-intervention in the intervention compared with the control - No effect at FUP			
	[32] Cadore et al., 2014	Multicomponent exercise intervention (high-speed resistance training, balance, and gait exercises, on muscle strength) Individual, at thenursing home, supervised by one experienced physical trainer	12 weeks	Usual care (mobility exercise)	Institutionalised	Fried	Frail	32 (24)	16 (11)	16 (13)	12 weeks	Intervention<br>Pre 0.76±0.07 m/sec<br>Post 0.80±0.08 m/sec<br>Control<br>Pre 0.68±0.06 m/sec<br>Post 0.60±0.07 m/sec No significant difference in change pre-post between intervention and control			
	[48] Wolf	Balance Training, individual, supervised by one instructor	15 weeks	Education + exercise suggestions	Community dwelling	biomedical, functional and psychosocial indicators	Prefrail	128 (104)	64 (50)	64 (54)	15 weeks + 4 months	Mean, kg (SD)<br>Intervention<br>Pre 24.87 (8.1)<br>Post 23.87 (8.0)<br>FUP 23.17 (8.0)<br>Control<br>Pre 23.87 (6.5)<br>Post 22.07 (6.2)<br>FUP 22.27 (6.6)<br>No significant difference in change between intervention and control.			

Handgrip strength	[48] Wolf	Tai Chi, group, supervised by one instructor	15 weeks	Education + exercise suggestions	Community dwelling	biomedical, functional and psychosocial indicators	Prefrail	136 (112)	72 (58)	64 (54)	15 weeks + 4 months	Mean, kg (SD)<br>Intervention<br>Pre 23.27 (8.29)<br>Post 22.57 (8.5)<br>FUP 22.87 (8.1)<br>Control<br>Pre 23.87 (6.5)<br>Post 22.07 (6.2)<br>FUP 22.27 (6.6)<br>Significant difference in change between intervention and control.
	[40] Kim Suzuki	Multicomponent exercise intervention, moderate intensity (warm-up, strengthening exercises, balance and gait training, cool-down).<br>Group, supervised (instructor + trainers), delivered at a research institute of gerontology	3 months	Usual care	Community dwelling	Fried	Frail	66 (63)	33 (31)	33 (32)	3 months (+ 4 months of follow up post intervention)	Mean, kg (SD)<br>Intervention<br>Pre 17.94 ± 3.00<br>Post 18.36 ± 3.28<br>Post 4 m 17.75 ± 2.90<br>Control<br>Pre 18.92 ± 3.38<br>Post 19.18 ± 3.50<br>Post 4 m 18.08 ± 2.92<br>No significant difference in mean change bewteen pre- and post-intervention in the intervention compared with the control - No effect at FUP
	[32] Cadore et al., 2014	Multicomponent exercise intervention (high-speed resistance training, balance, and gait exercises, on muscle strength) Individual, at thenursing home, supervised by one experienced physical trainer	12 weeks	Usual care (mobility exercise)	Institutionalized	Fried	Frail	32 (24)	16 (11)	16 (13)	12 weeks	Mean, N (SD) Intervention<br>Pre 165±63<br>Post 183±52<br>Control<br>Pre 157±64<br>Post 130±58<br>Significant difference in change pre-post between intervention and control

Q1.2 Should nutritional interventions (e.g. supplementation, diet modification) be recommended to prevent or delay the progression of or to revert frailty?

OUTCOME	Study	Intervention	Duration of intervention (total)	Comparison	Setting	Frailty definition	Frail - Prefrail	Pt n (total)	Pt n (intervention)	Pt n (control)	FUP duration	Narrative description of results	Considering the evidence on the impact on Frailty as a whole (regardless of the definition used for the outcome)<br>Quality of evidence (GRADE) - overall	Quality of evidence (GRADE) - Prefrail	Quality of evidence (GRADE) - Frail
**Frailty according to a composite index (continuous)**	[45] Ng	Nutritional supplement designed to augment caloric intake by about 20% and provide about one third of the recommended daily allowances of vitamins and minerals administrated nurse	6 months	Usual care	Community dwelling	Fried (CHS)	Prefrail	99	49	50	1 year	M (SD)<br>Intervention<br>Pre 2.1 (0.78)<br>3 m 1.5 (1.06)<br>6 m 1.4 (0.78)<br>12 m 1.5 (0.91)<br>Control<br>Pre 1.8 (0.80)<br>3 m 1.3 (0.85)<br>6 m 1.4 (1.06)<br>12 m 1.6 (0.97)<br>Significant difference in change between intervention and control at 12 m.			
**Frailty according to a composite index (dichotomous - transition to a less severe frailty category)**	[40] Kim Suzuki	milk fat globule membrane (MFGM) supplementation	3 months	Usual care	Community dwelling	Fried	Frail	65 (62)	32 (30)	33 (32)	3 months (+ 4 months of follow up post intervention)	Frailty reversal<br>post intervention:<br>interv: 28.1%<br>contr: 30.3%<br>OR 0.90 (0.31–2.62)<br>post 4 m:<br>interv: 25.0%<br>contr: 30.3%<br>OR 1.87 (0.54–6.47)			
	[45] Ng	Nutritional supplement designed to augment caloric intake by about 20% and provide about one third of the recommended daily allowances of vitamins and minerals administrated nurse	6 months	Usual care	Community dwelling	Fried (CHS)	Prefrail	99	49	50	1 year	Transition to a lower frailty category<br>interv: 35.6%<br>contr: 15.2%<br>OR 2.98 (1.10-8.07)			
**Short Physical Performance Battery**	[41] Kim Lee	Nutritional supplementation (Protein-energy)	12 weeks	Usual care	Community dwelling	Presence of low mobility (usual gate speed < 0.6 m/second) and poor nutrition (Mini Nutritional Assessment score < 24)	Frail	87 (84)	43 (41)	44 (43)	12 weeks	Median or mean ± SD<br>Intervention<br>Pre 5.5 (1.5)<br>Post 5.8 (1.6)<br>Difference 0 (−1, 1)<br>Control<br>Pre 5.7 (1.8)<br>Post 5.4 (2.2)<br>Difference −1 (−1, 1)<br>Significant difference in mean change bewteen pre- and post-intervention in the intervention compared with the control			
**Time up and Go**	[40] Kim Suzuki	milk fat globule membrane (MFGM) supplementation	3 months	Usual care	Community dwelling	Fried	Frail	65 (62)	32 (30)	33 (32)	3 months (+ 4 months of follow up post intervention)	Intervention<br>Pre 10.77 ± 2.58<br>Post 10.53 ± 2.77<br>Post 4 m 7.76 ± 1.52<br>Control<br>Pre 10.44 ± 3.79<br>Post 10.00 ± 4.32<br>Post 4 m 7.99 ± 3.79<br>No significant difference in mean change bewteen pre- and post-intervention in the intervention compared with the control - No effect at FUP			
	[41] Kim Lee	Nutritional supplementation (Protein-energy)	12 weeks	Usual care	Community dwelling	Presence of low mobility (usual gate speed < 0.6 m/second) and poor nutrition (Mini Nutritional Assessment score < 24)	Frail	87 (84)	43 (41)	44 (43)	12 weeks	Median or mean ± SD [sec]<br>Intervention<br>Pre 22.2 ± 12.4<br>Post 21.4 ± 12.2<br>Difference −1.1 (−5.5, 1.9)<br>Control<br>Pre 21.5 ± 12.7<br>Post 26.4 ± 25.3<br>Difference 0.9 (−2.3, 4.5)<br>Significant difference in mean change bewteen pre- and post-intervention in the intervention compared with the control			
**Walking Speed**	[40] Kim Suzuki	milk fat globule membrane (MFGM) supplementation	3 months	Usual care	Community dwelling	Fried	Frail	65 (62)	32 (30)	33 (32)	3 months (+ 4 months of follow up post intervention)	Intervention<br>Pre 1.10 ± 0.22<br>Post 1.08 ± 0.23<br>Post 4 m 1.11 ± 0.20<br>Control<br>Pre 1.18 ± 0.24<br>Post 1.13 ± 0.22<br>Post 4 m 1.18± 0.23 No significant difference in mean change bewteen pre- and post-intervention in the intervention compared with the control - No effect at FUP	⊕⊕⊖⊖<br>**LOW**<br>**Due to study limitations, inconsistency (different populations and interventions), and imprecision**	⊕⊕⊖⊖<br>**LOW**<br>**(only one quite small study)**	⊕⊕⊖⊖<br>**LOW**<br>**only 2 quite small studies with some study limitations, imprecision**
	[41] Kim Lee	Nutritional supplementation (Protein-energy)	12 weeks	Usual care	Community dwelling	Presence of low mobility (usual gate speed < 0.6 m/second) and poor nutrition (Mini Nutritional Assessment score < 24)	Frail	87 (84)	43 (41)	44 (43)	12 weeks	Median or mean ± SD [m/sec]<br>Intervention<br>Pre 0.35 ± 0.13<br>Post 0.35 ± 0.13<br>Difference 0 (−0.06, 0.07)<br>Control<br>Pre 0.38 ± 0.13<br>Post 0.32 ± 0.14<br>Difference −0.04 (−0.13, 0.04)<br>Significant difference in mean change bewteen pre- and post-intervention in the intervention compared with the control			
	[45] Ng	Nutritional supplement designed to augment caloric intake by about 20% and provide about one third of the recommended daily allowances of vitamins and minerals administrated nurse	6 months	Usual care	Community dwelling	Fried (CHS)	Prefrail	99	49	50	1 year	M (SD)<br>Intervention<br>Pre 5.8 (1.81)<br>3 m 4.8 (1.21)<br>6 m 5.0 (1.02)<br>12 m 5.2 (1.21)<br>Control<br>Pre 5.6 (2.07)<br>3 m 5.1 (2.09)<br>6 m 4.9 (1.47)<br>12 m 5.2 (1.72)<br>No significant difference in change between intervention and control at any time			
**Handgrip strength**	[40] Kim Suzuki	milk fat globule membrane (MFGM) supplementation	3 months	Usual care	Community dwelling	Fried	Frail	65 (62)	32 (30)	33 (32)	12 weeks	Median or mean ± SD [kg]<br>Intervention<br>Pre 15.3 ± 4.6<br>Post 15.1 ± 4.8<br>Difference 0.5 (−2, 2)<br>Control<br>Pre 16.3 ± 5.0<br>Post 16.4 ± 5.3<br>Difference −1 (−2, 2)<br>No significant difference in mean change bewteen pre- and post-intervention in the intervention compared with the control			
	[41] Kim Lee	Nutritional supplementation (Protein-energy)	12 weeks	Usual care	Community dwelling	Presence of low mobility (usual gate speed < 0.6 m/second) and poor nutrition (Mini Nutritional Assessment score < 24)	Frail	87 (84)	43 (41)	44 (43)	12 weeks	Median or mean ± SD [kg]<br>Intervention<br>Pre 15.3 ± 4.6<br>Post 15.1 ± 4.8<br>Difference 0.5 (−2, 2)<br>Control<br>Pre 16.3 ± 5.0<br>Post 16.4 ± 5.3<br>Difference −1 (−2, 2)<br>No significant difference in mean change bewteen pre- and post-intervention in the intervention compared with the control			

Q1.3 Should exercise combined with nutritional interventions be recommended to prevent or delay the progression of or to revert frailty?

													Considering the evidence on the impact on Frailty as a whole (regardless of the definition used for the outcome)		
**OUTCOME**	**Study**	**Intervention**	**Duration of intervention (total)**	**Comparison**	**Setting**	**Frailty definition**	**Frail - Prefrail**	**Pt n (total)**	**Pt n (intervention)**	**Pt n (control)**	**FUP duration**	**Narrative description of results**	**Quality of evidence (GRADE) - overall**	**Quality of evidence (GRADE) - Prefrail**	**Quality of evidence (GRADE) - Frail**
**Frailty according to a composite index (continuous)**	No study												⊕⊕⊖⊖ **LOW only 2 quite small studies with some study limitations, inconsistency (quite different interventions), imprecision**	**NA**	⊕⊕⊖⊖ **LOW only 2 quite small studies with some study limitations, inconsistency (quite different interventions), imprecision**
**Frailty according to a composite index (dichotomous - transition to a less severe frailty category)**	[40] Kim Suzuki	Multicomponent exercise intervention, moderate intensity (warm-up, strengthening exercises, balance and gait training, cool-down). Group, supervised (instructor + trainers), delivered at a research institute of gerontology PLUS Milk fat globule membrane (MFGM) supplementation	3 months	Usual care	Community dwelling	Fried	Frail	66 (65)	33 (33)	33 (32)	3 months (+ 4 months of follow up post intervention)	Reversal post intervention: interv: 57.3% contr: 30.3% OR 3.12 (1.13–8.60) post 4 m: interv: 45.5% contr: 15.2% OR 4.67 (1.45–15.08)			
**Short Physical Performance Battery**	No study														
**Time up and Go**	[31] Bonnefoy	Home based exercise (alone) PLUS dietary protein supplementation	4 months	Usual care	Community dwelling	score 3-6 on CCSHA-CFS-TV and then by score ≥ 1 on CHS-PCF	Frail	102 (96)	53	49	4 months	Variation % (1st Q, 3rd Q) Intervention 5.45 (-17.2; 13.2) Control 0 (-11.5; 15.5) No significant difference in change pre-post between intervention and control			
	[40] Kim Suzuki	Multicomponent exercise intervention, moderate intensity (warm-up, strengthening exercises, balance and gait training, cool-down). Group, supervised (instructor + trainers), delivered at a research institute of gerontology PLUS Milk fat globule membrane (MFGM) supplementation	3 months	Usual care	Community dwelling	Fried	Frail	66 (65)	33 (33)	33 (32)	3 months (+ 4 months of follow up post intervention)	Intervention Pre 9.63 ± 2.15 Post 7.98 ± 1.44 Post 4 m 6.93 ± 1.61 Control Pre 10.44 ± 3.79 Post 10.00 ± 4.32 Post 4 m 7.99 ± 3.79 Significant difference in mean change bewteen pre- and post-intervention in the intervention compared with the control - No effect at FUP			
**Walking Speed**	[31] Bonnefoy	Home based exercise (alone) PLUS dietary protein supplementation	4 months	Usual care	Community dwelling	score 3-6 on CCSHA-CFS-TV and then by score ≥ 1 on CHS-PCF	Frail	102 (96)	53	49	4 months	Variation % (1st Q, 3rd Q) Intervention 0 (-13.7; 16.1) Control 0 (-19.5; 15.9) No significant difference in change pre-post between intervention and control			
	[40] Kim Suzuki	Multicomponent exercise intervention, moderate intensity (warm-up, strengthening exercises, balance and gait training, cool-down). Group, supervised (instructor + trainers), delivered at a research institute of gerontology PLUS Milk fat globule membrane (MFGM) supplementation	3 months	Usual care	Community dwelling	Fried	Frail	66 (65)	33 (33)	33 (32)	3 months (+ 4 months of follow up post intervention)	Intervention Pre 1.15 ± 0.16 Post 1.25 ± 0.24 Post 4 m 1.23 ± 0.21 Control Pre 1.18 ± 0.24 Post 1.13 ± 0.22 Post 4 m 1.18± 0.23 Significant difference in mean change bewteen pre- and post-intervention in the intervention compared with the control - No effect at FUP			

**Handgrip strength**	[40] Kim Suzuki	Multicomponent exercise intervention, moderate intensity (warm-up, strengthening exercises, balance and gait training, cool-down). Group, supervised (instructor + trainers), delivered at a research institute of gerontology PLUS Milk fat globule membrane (MFGM) supplementation	3 months	Usual care	Community dwelling	Fried	Frail	66 (65)	33 (33)	33 (32)	3 months (+ 4 months of follow up post intervention)	Median or mean ± SD [kg] Intervention Pre 17.19 ± 3.79 Post 17.83 ± 4.05 Post 4 m 17.00 ± 3.88 Control Pre 18.92 ± 3.38 Post 19.18 ± 3.50 Post 4 m 18.08 ± 2.92 No significant difference in mean change bewteen pre- and post-intervention in the intervention compared with the control - No effect at FUP

OUTCOME	Intervention category	Intervention sub-category	Study	Intervention - description	Duration of intervention (total)	Comparison	Setting	Frailty definition	Frailty criteria	Frail - Prefrail	Pt n (total)	Pt n (intervention)	Pt n (control)	FUP duration	Narrative description	Quality of evidence (GRADE) - overall	Quality of evidence (GRADE) - Prefrail	Quality of evidence (GRADE) - Frail
**Frailty according to a composite index (continuous)**	2. uni-professional	Active Management (mainly) and (Psycho)educational - Only health care professionals - At home - Individual	[46] Van Hout	Nurse home visits	at least 4 visits a year	Usual Care	community dwelling	composite of biomedical, functional and psychosocial indicators (based on COOP-WONCA charts)	≥ 2 of 6 COOP-WONCA charts	Frail	651	331	320	18 months (and 6 months)	Effect size on COOP-WONCA-based Frailty not reported. They report SF-36 mean scores at baseline and FUP. They qualitative report a non statistically significant difference between intervention and control (non signficant groupxtime interaction)			
	2. uni-professional	(Psycho)educational (mainly) and Active Management - Only health care professionals - At home (but not clear) - Individual	[47] Vriendt	Client-centred, activity-oriented and community based intervention program, delivered by trained occupational therapists	8 to 10 weeks	Usual Care	community dwelling/primary care	based on b-ADL functioning (BEL-profile scale)	≥ 1 impairment	Frail	168	86 (82)	82 (80)	8-10 weeks	Mean difference (SD) between intervention and control in b-ADL scale change pre-post (b-ADL scores expressed as a percentage where 0% represented complete dependency and 100% complete independence) 6.7 (1.4 to 12.1), p = 0.013			
**Frailty according to a composite index (dichotomous - transition to a less severe frailty category)**	2. uni-professional	(Psycho)educational - Not only health care professionals - At home - Individual	[30] Behm [38] Gustafsson	single preventive home visit (1.5-2 h) made by an occupational therapist (OT), a physiotherapist (PT), a registered nurse (RN), **or** a qualified social worker (SW)	each home visit: 1.5-2 h	Usual care	community dwelling	1. Measured as sum of 8 (Behm) or 6 (Gustafsson) biomedical, functional and psychosocial indicators; 2. Measured as tiredness in daily activities (Mob-T scale)	1. ≥ 3 positive indicators	13% non- frail 68% prefrail 19% frail	288	174	114	2 y	Deterioration in Frailty measured as ***sum of indicators,*** between Baseline and follow-up time<br>**3 months** % of No deterioration in Frailty n (%)<br>Control 81 (71); Intervention 121 (70)<br>OR (95 % CI) 0.93 (0.55–1.56)<br>**1 year** % of deterioration<br>control 39 %; intervention 34 %<br>OR (95 CI) 0.79 (0.49–1.28)<br>**2 year** % of deterioration<br>control 59 %; intervention 52 %<br>OR (95 CI) 0.77 (0.48–1.24)<br>BUT: Significant effect on deterioration in Frailty measured as ***Tiredness*** (with a lower rate of deterioration in intervetion compared with control group) at 1 year (not at 2 year)	**⊕⊕⊖⊖ LOW Due to study limitations, inconsistency (different populations and interventions), imprecision**	**⊕⊕⊖⊖ LOW (only one study)**	**⊕⊕⊖⊖ LOW Due to substantial study limitations, inconsistency (different interventions), imprecision**
	2. uni-professional	(Psycho)educational (mainly) and Active Management - Only health care professionals - At home - Individual	[36] Favela	Nurse home visits	weekly visits over 9 months	Usual Care	community dwelling	1. 34-variable Frailty Index (Rookwood) 2. Fried	1. ≥ 0.14 in Frialty index 2. ≥ 3 criteria	All Frail according to Rookwood) - about 45% frail according to Fried	88	44	44	9 months	% Reversal from Frailty (Fried) [data in part derived from a graph]<br>Intervention about 11%<br>Control about 10%<br>% Development of Frailty<br>Intervention about 24.3%<br>Control about 12.8%			
	2. uni-professional	(Psycho)educational (mainly) and Active Management - Only health care professionals - At home - Individual	[36] Favela	Nurse home visits alone with alert buttons	weekly visits over 9 months	Usual Care	community dwelling	1. 34-variable Frailty Index (Rookwood) 2. Fried	1. ≥ 0.14 in Frialty index 2. ≥ 3 criteria	All Frail according to Rookwood) - about 45% frail according to Fried	89	45	44	9 months	% Reversal from Frailty (Fried) [data in part derived from a graph]<br>Intervention 12.8%<br>Control about 10%<br>% Development of Frailty<br>Intervention about 5.1%<br>Control about 12.8%			

Q2.2 Should MULTI-PROFESSIONAL interventions based on tailored care/GEM be recommended to prevent or delay the progression of or to revert frailty?

OUTCOME	Intervention category	Intervention sub-category	Study	Intervention - description	Duration of intervention (total)	Comparison	Setting	Frailty definition	Frailty criteria	Frail - Prefrail	Pt n (total)	Pt n (intervention)	Pt n (control)	FUP duration	Narrative description	Considering the evidence on the impact on Frailty as a whole (regardless of the definition used for the outcome)<br>Quality of evidence (GRADE) - overall	Quality of evidence (GRADE) - Prefrail	Quality of evidence (GRADE) - Frail
**Frailty according to a composite index (dichotomous - transition to a less severe frailty category)**	1. multi-professional	(Psycho)educational - Not only health care professionals - At home - Group based and Individual	[30] Behm<br>[38] Gustafsson	multi-professional senior group meetings with one follow-up home visit occupational therapist (OT), a physiotherapist (PT), a registered nurse (RN), **or** a qualified social worker (SW)	6-7 w	Usual care	community dwelling	1. Measured as sum of 8 (Behm) or 6 (Gustafsson) biomedical, functional and psychosocial indicators; 2. Measured as tiredness in daily activities (Mob-T scale)	1. ≥ 3 positive indicators	13% non- frail<br>68% prefrail<br>19% frail	285	171	114	2 y	Deterioration in Frailty measured as ***sum of indicators,*** between Baseline and follow-up time<br>**3 months** % of No deterioration in Frailty n (%)<br>Control 81 (71); Intervention 110 (64)<br>OR (95 % CI) 0.73 (0.44–1.23)<br>**1 year** % of deterioration<br>control 39 %; intervention 34 %<br>OR (95 CI) 0.79 (0.48–1.29)<br>**2 year** % of deterioration<br>control 59 %; intervention 47 %<br>OR (95 CI) 0.63 (0.39–1.02)<br>BUT: Significant effect on deterioration in Frailty measured as ***Tiredness*** (with a lower rate of deterioration in intervetion compared with control group) at 1 year (not at 2 year)	⊕⊕⊖⊖<br>**LOW**<br>**Due to study limitations, inconsistency (different populations and interventions), imprecision**	⊕⊕⊖⊖ **LOW**<br>**Due to study limitations, inconsistency (different populations and interventions), imprecision**	⊕⊕⊖⊖<br>**LOW**<br>**Due to study limitations, inconsistency (different populations and interventions), imprecision**
	1. multi-professional	Active management - Not only health care professionals - At home + community hospital - Individual	[42] Li	Screening evaluation based on CGA by trained nurses + appropriate intervention based on screening results (CGA report revised by two geriatricians; intervention programs delivered by medical professionals, but involving other professionals including social workers)	not specified	Screening evaluation based on CGA only	community hospital	Fried	≥ 3 criteria	pre-frail or frail (mostly pre-frail)	310	152 (129)	158 (140)	6 months	% Deterioration in Frailty status<br>Intervention 8.5%<br>Control 10.7%<br>OR (95% CI) 0.78 (0.34–1.79)<br>% Improvement in Frailty status<br>Intervention 4.6%<br>Control 1.4%<br>OR (95% CI) 0.94 (0.42–2.12)			
	1. multi-professional	Active management - Not only health care professionals - from the ED, across different settings of care - Individual	[23] Eklund	Individual, provided by professionals in nursing with geriatric competence (emergency department), occupational therapy, physiotherapy and social work (municipality), no geriatrician	1 y	Usual care	patients discharged from the ED	composite of biomedical, functional and psychosocial indicators	≥ 2 positive indicators among the ones prespecified	Mostly frail and pre-frail (non frail only 5 % in the intervention and 0 % in the control 0 5 24 26 76 69, respectively)	161 (181 randomized)	85	76	1 y	Improvement in Frailty measure % (n)<br>**3 month**<br>intervention 8 (7)<br>control 13 (10)<br>OR (95 % CI) 0.59 (0.21-1.64)<br>**6 month**<br>intervention 12 (10)<br>control 17 (13)<br>OR (95 % CI) 0.65 (0.27-1.57)<br>**12 month**<br>intervention 12 (10)<br>control 22 (17)<br>OR (95 % CI) 0.46 (0.20-1.09)			
	1. multi-professional	Active management - Only health care professionals - At home - Individual	[26] Fairhall	Individual, provided by two physiotherapists, a geriatrician, rehabilitation physician, dietician, and nurse, GEM based	1 y	Usual care	community dwelling	Fried	≥ 3 criteria	frail	241	120	121	1 y	**3 month**<br>n Frail (%)<br>Intervention 71 (64); Control 88 (75)<br>Difference Between Groups, Adjusted for Month 0. Intervention Minus Control (95% CI, P Value)<br>-11.3% (-23.3% to -0.7%, P 0 .07)<br>**12 month**<br>n Frail (%)<br>Intervention 66 (62); Control 84 (77)<br>Difference Between Groups, Adjusted for Month 0. Intervention Minus Control (95% CI, P Value)<br>-14.7% (-27.0% to -2.4%, P 0 .02)			
	1. multi-professional	Active management - Only health care professionals - At the primary care centre - Individual	[43] Monteserin	individual CGA + group based recommendations by a trained nurse about healthy habits and adherence to treatment (if not at risk of frailty) + Individual based sessions with geriatrician (if at risk of frailty)	group session with the nurse: 45 mins; individual sessions with the geriatrician 30 mins	Usual care	community dwelling/primary care	composite of biomedical, functional and psychosocial indicators	≥ 2 positive indicators among the ones prespecified	at risk of frailty	620	308 (157 not at risk of frailty; 151 at risk of frailty)	312	18 months	% Reversal from *at risk of frailty* to *not at risk of frailty*<br>Control 13.5%; Intervention 27.9%)<br>Adjusted OR (95% CI) for reversal 3.08 (1.21–7.82)<br>% Progression from *not at risk of frailty* to *at risk of frailty*<br>Control 33.8% Intervention 20.4% (p=0.0027)			
**Other composite measures of Frailty as Physical Performance (continuous)**	1. multi-professional	Active management - Not only health care professionals - at hospital discharge - Individual	[35] Cohen	Outpatient care in a GEM unit, individual-based, provided by a geriatrician, a social worker, and a nurse - using Preventive and management services (e.g., dietetics, physical and occupational therapy, and clinical pharmacy)	1 y	Usual care	inpatients (once discharged)	composite of functional and psychosocial indicators	≥ 2 positive indicators among the ones prespecified	frail	1388	692	696	1 y	mean changes in the Physicial Performance Test scores (adjusted for the length of stay)<br>At discharge<br>Intervention 2.34<br>Control 2.60<br>P 0.24<br>12 month<br>Intervention 4.67 (2.13)<br>Control 4.07 (1.30)<br>P 0.12			
	1. multi-professional	Active management - Not only health care professionals - In hospital - Individual	[35] Cohen	Inpatient care in a GEM unit, individual-based, provided by a geriatrician, a social worker, and a nurse - using Preventive and management services (e.g., dietetics, physical and occupational therapy, and clinical pharmacy)	30 days	Usual care	inpatients	composite of functional and psychosocial indicators	≥ 2 positive indicators among the ones prespecified	frail	1388	694	694	1 y	mean changes in the Physicial Performance Test scores (adjusted for the length of stay)<br>At discharge<br>Intervention 3.12<br>Control 1.75<br>P <0.001<br>At 12 month<br>Intervention 4.50<br>Control 4.24<br>P 0.51			

Q3 Should *other types of interventions* be recommended to prevent or delay the progression of or to revert frailty?

Intervention group	Intervention subgroup	Study	Intervention description	Duration of intervention (total)	Comparison	Setting	Frailty definition	Frail - Prefrail	Pt n (total)	Pt n (intervention)	Pt n (control)	FUP duration	OC Frailty - continuous	OC Frailty - dichotomous	OC TUG	OC Walking speed	OC grip strenght	OC SPPB	Comment	**Quality of evidence**
Other	Cognitive training	[45] Ng	Activities designed to stimulate short-term memory, and enhance attention and information-processing skills, and reasoning and problem solving abilities in the first 12 weeks.<br>In the subsequent 12 weeks "booster" sessions, focusing on the revision of the cognitive skills learned.	6 months	Usual care	Community dwelling	Fried	Prefrail	98	48	50	1 year	M (SD)<br>Intervention<br>Pre 2.0 (0.91)<br>3 m 1.3 (0.81)<br>6 m 1.4 (0.78)<br>12 m 1.4 (0.94)<br>Control<br>Pre 1.8 (0.80)<br>3 m 1.3 (0.85)<br>6 m 1.4 (1.06)<br>12 m 1.6 (0.97)<br>Significant difference in change between intervention and control at 12 m.	Transition to a lower frailty category<br>interv: 35.6%<br>contr: 15.2%<br>OR 2.89 (1.07-7.82)	.	M (SD) [sec]<br>Intervention<br>Pre 5.4 (1.16)<br>3 m 4.7 (0.97)<br>6 m 4.6 (0.80)<br>12 m 5.2 (1.05)<br>Control<br>Pre 5.6 (2.07)<br>3 m 5.1 (2.09)<br>6 m 4.9 (1.47)<br>12 m 5.2 (1.72)<br>No significant difference in change between intervention and control at any time	.	.		see Table 3 of *FOCUS D4.1.2 Systematic Review: A systematic review of the effectiveness of frailty interventions* for methodological appraisal of studies included
Physical	Exercise + nutrition + cognitive training	[45] Ng	Multicomponent exercise intervention, moderate, gradually increasing intensity, tailored to participants' individual abilities. Resistance exercises, balance training exercises involving functional strength, sensory input, and added attentional demands. Group, supervised by a qualified trainer (for 12 weeks) then alone<br>PLUS Nutritional supplement designed to augment caloric intake by about 20% and provide about one third of the recommended daily allowances of vitamins and minerals<br>administrated nurse<br>PLUS cognitive training	6 months	Usual care	Community dwelling	Fried	Prefrail	98	48	50	1 year	M (SD)<br>Intervention<br>Pre 2.1 (0.81)<br>3 m 1.3 (0.84)<br>6 m 1.4 (0.87)<br>12 m 1.2 (1.07)<br>Control<br>Pre 1.8 (0.80)<br>3 m 1.3 (0.85)<br>6 m 1.4 (1.06)<br>12 m 1.6 (0.97)<br>Significant difference in change between intervention and control at 3m, 6 m and 12 m.	Transition to a lower frailty category<br>interv: 47.8%<br>contr: 15.2%<br>OR 5.00 (1.88-13.3)	.	M (SD) [sec]<br>Intervention<br>Pre 5.4 (1.25)<br>3 m 4.7 (1.20)<br>6 m 4.8 (1.13)<br>12 m 5.3 (2.17)<br>Control<br>Pre 5.6 (2.07)<br>3 m 5.1 (2.09)<br>6 m 4.9 (1.47)<br>12 m 5.2 (1.72)<br>No significant difference in change between intervention and control at any time	.	.		see Table 3 of *FOCUS D4.1.2 Systematic Review: A systematic review of the effectiveness of frailty interventions* for methodological appraisal of studies included
Other	Hormone therapy	[44] Muller	atamestane	36 w	Placebo	Community dwelling	Physicial indicators (isometric grip strength <30 kg and leg extensor power <100 Nm)	Prefrail (not explicitely defined as such)	49	25	24	36 w	.	.	.	.	Mean change from baseline in placebo group (CI 95%) [kg]:<br>1.2 (2.4; 0.0)<br>Difference between placebo and study agent<br>(95 % CI)<br>0.2 (- 1.8; 2.1)	.	only men, aged ≥ 70	see Table 3 of *FOCUS D4.1.2 Systematic Review: A systematic review of the effectiveness of frailty interventions* for methodological appraisal of studies included
Other	Hormone therapy	[44] Muller	DHEA	36 w	Placebo	Community dwelling	Physicial indicators (isometric grip strength <30 kg and leg extensor power <100 Nm)	Prefrail (not explicitely defined as such)	49	25	24	36 w	.	.	.	.	Mean change from baseline in placebo group (CI 95%) [kg]:<br>1.2 (2.4; 0.0)<br>Difference between placebo and study agent beta (95 % CI)<br>1.3 (- 0.6; 3.2)	.	only men, aged ≥ 70	see Table 3 of *FOCUS D4.1.2 Systematic Review: A systematic review of the effectiveness of frailty interventions* for methodological appraisal of studies included
Other	Hormone therapy	[44] Muller	atamestane + DEHA	36 w	Placebo	Community dwelling	Physicial indicators (isometric grip strength <30 kg and leg extensor power <100 Nm)	Prefrail (not explicitely defined as such)	50	26	24	36 w	.	.	.	.	Mean change from baseline in placebo group (CI 95%) [kg]:<br>1.2 (2.4; 0.0)<br>Difference between placebo and study agent beta (95 % CI)<br>0.0 (- 1.9; 1.9)	.	only men, aged ≥ 70	see Table 3 of *FOCUS D4.1.2 Systematic Review: A systematic review of the effectiveness of frailty interventions* for methodological appraisal of studies included
Other	Exercise + nutritional consultation	[33] Chan	Multicomponent exercise intervention (stretching, resistance training, postural control and balance training). Group (?), supervised (?), performed at the participating hospital<br>During exercise sessions partiicants received nutritional consultations and were then followed-up over teh phone to assess compliance with dietary advice	3 times a week for 3 months	No intervention (educational booklet provided to both intervention and control group)	Community dwelling	Chinese Canadian Study of Health and Aging Clinical Frailty Scale Telephone Version (CCSHA_CFS_TV) combined with modified Fried criteria	87% prefrail<br>13% frail	117	55	62	12 mo		% Improvement in Frailty<br>Control<br>3 mo 27%<br>6 mo 26%<br>12 mo 31%<br>Intervention<br>3 mo 45%<br>6 mo 42%<br>12 mo 40%<br>Significant difference between intervention and control only in improvement from baseline to **3 mo** (p=0.008)						see Table 3 of *FOCUS D4.1.2 Systematic Review: A systematic review of the effectiveness of frailty interventions* for methodological appraisal of studies included
Other	Problem Solving Therapy	[33] Chan	Sessions of psychotherapy to solve the "here-and-now" problems contributing to their mood-related conditions and helps increase their self-efficacy, led by case managers	6 sessions for 3 months	No intervention (educational booklet provided to both intervention and control group)	Community dwelling	Chinese Canadian Study of Health and Aging Clinical Frailty Scale Telephone Version (CCSHA_CFS_TV) combined with modified Fried criteria	87% prefrail<br>13% frail	117	57	60	12 mo		Control<br>3 mo 28%<br>6 mo 32%<br>12 mo 35%<br>Intervention<br>3 mo 44%<br>6 mo 35%<br>12 mo 35%<br>No significant difference between intervention and						see Table 3 of *FOCUS D4.1.2 Systematic Review: A systematic review of the effectiveness of frailty interventions* for methodological appraisal of studies included
