# Supplementary material for: Interventions to prevent, delay or reverse frailty in older people: a journey towards clinical guidelines
Source: BMC Med. 2019 Oct 29;17:193. doi: 10.1186/s12916-019-1434-2 (PMC6819620; doi:10.1186/s12916-019-1434-2)
Supplement: Supplementary file 2 — Additional file 2. Narrative synthesis of the effect of interventions on secondary outcomes, as offered to the guideline panellists. [file 12916_2019_1434_MOESM2_ESM.pdf]

FOCUS guidelines: narrative summary of quantitative evidence on the effect of interventions on secondary outcomes as collected in the FOCUS Systematic Review and presented to the panellists.

Q1.1 Should interventions based on physical activity/exercise be recommended to prevent or delay the progression of or to revert frailty?

Different types of physical activity programmes (i.e. multicomponent exercise programmes, either individual or group-based, tai chi, and the programme based on Jaques-Dalcroze eurhythmics) were shown to reduce the incidence of and/or injury associated with falls.

The effect of physical interventions on function (i.e. the ability to perform activities of daily living, either basic or instrumental) was heterogeneous.

Only one study (Ng et al.) explored the effect of either an exercise programme alone or in combination with nutritional supplementation on hospitalisation over 12 months, and did not find any statistically significant difference compared with usual care.

In the studies included in the FOCUS D4.1.2 Systematic Review: A systematic review of the effectiveness of frailty interventions there are no outcomes specifically explored as ***safety* outcomes**. Please simply consider the overall evidence table for each question.

Q2 - Should interventions based on tailored care and/or Geriatric Evaluation and Management (GEM) be recommended to prevent or delay the progression of frailty, or to revert frailty?

Given the nature of interventions in Q2, studies included the secondary outcomes of interest more likely than in case of studies on physical interventions, but effects they found were sparse.

In one study (Cohen et al.), neither a GEM-based intervention on inpatients nor a GEM-based intervention on outpatients had a significant effect on mortality, compared with usual care. Conversely, in Monteserin et al., a multi-professional GEM-intervention based on a group session with the nurse combined with an individual session with a geriatrician for those at risk of frailty, reduced the composite outcome of all causes of death, admissions to nursing home facilities and admissions to a home care programme. In Van Hout et al., home visits made by nurses were associated with more accesses to hospital than usual care (likely because of a tighter monitoring and problem finding).

A greater effect on ability to perform activities of daily living, compared with control was found for multi-professional GEM-based interventions, on either inpatients (Cohen et al.) or patients at discharge form the ED (Eklund et al.). An effect was found also for multi-professional senior group meetings, compared with usual care and with an alternative intervention based on a single uni-professional home visit (Gustafsson et al.), but not in the case of a GEM-based intervention delivered in a community hospital (Li et al.).

Few studies explored the effect on quality of life. Fairhall et al. found no effect of a multi-professional GEM-based programme (EQ-5D). Gustafsson et al. found a significant effect associated both with multi-professional senior group meetings and with the intervention based on a single uni-professional home visit (self-rated health).

In Cohen et al., the GEM-based interventions on inpatients and outpatients was associated with a smaller decrease, on average, of the score on the general health component of the SF-36 tool at discharge (inpatient intervention) and at 12 months (outpatient interventions) compared with usual care. The GEM-based intervention on outpatients was associated with an larger increase, on average, of the score on the mental health component of the of the SF-36 tool, compared with usual care.

In the studies included in the FOCUS D4.1.2 Systematic Review: A systematic review of the effectiveness of frailty interventions there are no outcomes specifically explored as ***safety* outcomes**. Please simply consider the overall evidence table for each question.

Q3 - Should "other interventions" be recommended to prevent or delay the progression of frailty, or to revert frailty?

In Chan et al., neither the exercise + nutritional consultation intervention nor the problem solving therapy was associated with a significant effect on cognitive and mental performance (Mini Mental State Examination, Primary Care Evaluation of Mental Disorders [PRIME-MD], function (Barthel Index), and quality of life (EQ-5D).

In Ng et al. neither the cognitive training alone nor its combination with the exercise programme and nutritional supplement had a significant effect on function, falls, and hospitalization.

In the studies included in the FOCUS D4.1.2 Systematic Review: A systematic review of the effectiveness of frailty interventions there are no outcomes specifically explored as ***safety* outcomes**. Please simply consider the overall evidence table for each question.
