# Supplementary material for: Interventions to prevent, delay or reverse frailty in older people: a journey towards clinical guidelines
Source: BMC Med. 2019 Oct 29;17:193. doi: 10.1186/s12916-019-1434-2 (PMC6819620; doi:10.1186/s12916-019-1434-2)
Supplement: Supplementary file 3 — Additional file 3: Table S1. Table that describes the members of the FOCUS Guideline Panel and their role in the development of the guidelines. [file 12916_2019_1434_MOESM3_ESM.pdf]

Table S1. The FOCUS guidelines panel

Panelist			Role in the guidelines development			
**Name (alphabetic order)**	**Institution and Country**	**Profile**	**Technical team - iEtD administrator**	**Evidence base generation and/or review**	**Voting**	**Revision of final guidelines**
João Apóstolo	Escola Superior de Enfermagem de Coimbra (ESEnfC), Portugal	Psychogeriatric nurse, Professor of Ageing		x	x	x
Elzbieta Bobrowicz-Campos	Escola Superior de Enfermagem de Coimbra (ESEnfC), Portugal	Specialist in clinical and health psychology		x	x	x
Maria Bujnowska-Fedak	Wroclaw Medical University (WMU), Poland	Family medicine specialist and geriatrician		x	x	x
Antonio Cano	Universitat de València (UVEG)	Professor of Obstetrics and Gynaecology, specialist in ageing of women		x	x	x
Richard Cooke	Aston Research Centre for Healthy Ageing, Aston University (ARCHA), UK	Health psychologist, Senior Lecturer		x		x
Barbara D'Avanzo	IRCCS Istituto di Ricerche Farmacologiche "Mario Negri", Italy	Senior researcher in mental health and ageing		x	x	x
Sarah Damanti	Fondazione IRCCS Cà Granda - Ospedale Maggiore Policlinico, Italy	Geriatrician, PhD candidate	x	x		x
Federico Germini	Fondazione IRCCS Cà Granda - Ospedale Maggiore Policlinico, Italy	Emergency medicine physician and clinical epidemiologist	x	x		x
Holly Gwyther	Aston Research Centre for Healthy Ageing, Aston University (ARCHA), England	Chartered psychologist specialising in ageing		x	x	x
Carol Holland	Centre for Ageing Research, Division of Health Research, Lancaster University, UK	Chartered psychologist, Professor of Ageing		x	x	x
Donata Kurpas	Wroclaw Medical University (WMU) , Poland	Family medicine specialist and public health specialist		x	x	x

Maura Marcucci	Fondazione IRCCS Cà Granda - Ospedale Maggiore Policlinico	General internist and clinical epidemiologist	x	x		x
Alessandro Nobili	IRCCS Istituto di Ricerche Farmacologiche "Mario Negri", Italy	Clinical pharmacologist of ageing		x	x	x
Silvina Santana	University of Aveiro (UAVR), Portugal	Health services management and research, Professor of Business Management Sciences		x	x	x
Rachel Shaw	Aston Research Centre for Healthy Ageing, Aston University (ARCHA), UK	Health Psychologist, Reader in Psychology		X		x
Katarzyna Szwamel	Wroclaw Medical University (WMU), Poland	Emergency nurse academic teacher, PhD candidate			x	x
