# Supplementary material for: Interventions to prevent, delay or reverse frailty in older people: a journey towards clinical guidelines
Source: BMC Med. 2019 Oct 29;17:193. doi: 10.1186/s12916-019-1434-2 (PMC6819620; doi:10.1186/s12916-019-1434-2)
Supplement: Supplementary file 4 — Additional file 4. Example of the output of the voting process using the Evidence to Decision template, as received and processed by the guidelines technical team. [file 12916_2019_1434_MOESM4_ESM.pdf]

ORIGINAL OUTPUT OF THE VOTING PROCESS

GQ - Should interventions to prevent or delay the progression of frailty, or to revert frailty, be adopted?

(Clinical recommendation - Population perspective)

COMMENTS

QUESTION

P1: There was some evidence comparing interventions reviewed in the systematic review, notably from Ng TP, Feng L, Nyunt MS, Feng L, Niti M, Tan BY, et al. Nutritional, physical, cognitive, and combination interventions and frailty reversal among older adults: A randomized controlled trial. Am J Med. 2015;128(11):1225-36. they compared physical exercise, nutrition and cognitive training and found advantages particularly for combination methods.

ASSESSMENT

P2: It is amazing that interventions giving evidence of effectiveness were eventually rather few. Some had very low quality which weakened the evidence. (DESIRABLE/UNDESIRABLE EFFECTS)

P1: this is a bit confusing as the question is about costs, I assume of the interventions, but the judgement is about costs and savings - it may have been better as two separate questions? (RESOURCES)

P2: We should remind that the evidence brought here from the qualitative studies, ie, the meta-synthesis and the focus groups with stakeholders, was related to the issue of how frailty was seen, and, in fact, the results are of a preminence of the mental and cognitive dimension over the physical one. This did not mean that the physical was not important, also because, as reported in the Additional considerations, physical frailty can cause isolation and cognitive frailty. In the focus groups, we also asked whether the possibility to make physical exercise was appreciated, and the answer was yes. Nonetheless, it is true that perceiving frailty as little preventable, can hinder motivation to make physical exercise. Personally, I see more a problem of accessibility. So, it

is fact mixed, and the indication could be that, according to the qualitative studies findings, physical interventions should be offered in a holistic framework, taking care of all the aspects, and overcoming the perspective of healthy ageing, which does not really challenge the idea of non-malleability of frailty. (VALUES)

P2: My view of the equity issue here is that services should be offered to all, which means helping those who have more difficulties access them. I do not see here a problem of equity in the interventions themselves. Nonetheless, from the qualitative studies emerged that there was a serious problem of poverty, and that interventions were more accepted by people of higher social classes. So, there is a structural problem of equity that should be addressed. I regret, from this point of view, that the equity indicator in the Delphi was one of the least valued. (EQUITY)

P1: Impact on health equity: a bit of a confusing question that could be misinterpreted: If I mean health equity is increased by frailty interventions, I'd put increased, but if I meant that the impact of frailty on health equity is reduced, I'd put reduced. I've gone with reduced, as in, health inequity is reduced by frailty interventions. (EQUITY)

P2: I have assumed that all these interventions are easily feasible. (FEASIBILITY)

SUMMARY OF JUDGEMENTS

								**DRAFT CONSENSUS JUDGEMNT**
Problem	- Don't know	- Varies		- No	- Probably No	1 Probably Yes	10 Yes	**Yes**
Desirable effects	- Don't know	- Varies		- Trivial	1 Small	8 Moderate	2 Large	**Moderate**
Undesirable effects	3 Don't know	- Varies		- Large	1 Moderate	2 Small	5 Trivial	**Small/Trivial – Don't know**
Certainty of the evidence	- No included studies			- Very low	8 Low	3 Moderate	- High	**Low**
Values				1 Important uncertainty or variability	4 Possibly important uncertainty or variability	5 Probably no important uncertainty or variability	1 No important uncertainty or variability	**Probably no important uncertainty or variability**

Balance of effects	- Don't know	- Varies	- Favours the comparison	1 Probably favours the comparison	1 Does not favour either the intervention or the comparison	4 Probably favours the intervention	5 Favours the intervention	**Probably favours the intervention**
Resources required	2 Don't know	- Varies	- Large costs	2 Moderate costs	2 Negligible costs or savings	1 Moderate savings	4 Large savings	**Moderate savings**
Certainty of evidence of required resources	1 No included studies			3 Very low	6 Low	1 Moderate	- High	**Low**
Cost-effectiveness	1 Don't know	- Varies	- Favours the comparison	- Probably favours the comparison	2 Does not favour either the intervention or the comparison	6 Probably favours the intervention	2 Favours the intervention	**Probably favours the intervention**
Equity	1 Don't know	- Varies	1 Reduced	- Probably reduced	- Probably no impact	8 Probably increased	1 Increased	**Probably increased**

Acceptability	-<br>Don't know	2<br>Varies		-<br>No	-<br>Probably No	7<br>Probably Yes	2<br>Yes	**Probably Yes**
Feasibility	-<br>Don't know	1<br>Varies		-<br>No	-<br>Probably No	8<br>Probably Yes	2<br>Yes	**Probably Yes**

Q1 - Should physical interventions be recommended to prevent or delay the progression of frailty, or to revert frailty?

COMMENTS

Undesirable effects:

P1: I have responded to this question as if it is asking what are the undesirable effects of frailty (therefore affecting my judgment as to whether interventions should be recommended. An alternative interpretation of the question could be how substantial are undesirable anticipated effects of conducting frailty interventions. My answers do not reflect an answer to that interpretation

Resources required:

P1: The evidence from the modelling is about benefits of intervention or costs of frailty, not about the costs of intervention

Certainty of evidence of required resources

P1: we don't have evidence on required resources yet

Acceptability:

P1: the group versus individual based recommendation is important. There is much more limited evidence for success of individual exercise programs, and so all my responses are positive towards interventions based on physical activity but with the provision that this is based on significant effects found for group based exercise. It may be useful to separate this out in the final guidelines

SUMMARY OF JUDGMENTS

- **Interventions based on exercise/physical activity**

						DRAFT CONSENSUS JUDGEMNT
Problem	5<br>Favours this option	1<br>Probably favours this option	1<br>Neither favours this option or other options	-<br>Probably favours other options	-<br>Favours other options	**Favours this option**
Desirable effects OVERALL	4<br>Favours this option	6<br>Probably favours this option	-<br>Neither favours this option or other options	-<br>Probably favours other options	-<br>Favours other options	**Probably favours this option (+)**
Desirable effects PREFRAIL	3<br>Favours this option	2<br>Probably favours this option	-<br>Neither favours this option or other options	-<br>Probably favours other options	-<br>Favours other options	**Favours this option (-)**
Desirable effects FRAIL	1<br>Favours this option	3<br>Probably favours this option	-<br>Neither favours this option or other options	-<br>Probably favours other options	-<br>Favours other options	**Probably favours this option (+)**
Undesirable effects OVERALL	2<br>Favours this option	4<br>Probably favours this option	3<br>Neither favours this option or other options	1<br>Probably favours other options	-<br>Favours other options	**Probably favours this option**

Undesirable effects PREFRAIL	1<br>Favours this option	2<br>Probably favours this option	1<br>Neither favours this option or other options	1<br>Probably favours other options	-<br>Favours other options	**Probably favours this option (-)**
Undesirable effects FRAIL	-<br>Favours this option	3<br>Probably favours this option	1<br>Neither favours this option or other options	1<br>Probably favours other options	-<br>Favours other options	**Probably favours this option**
Certainty of the evidence OVERALL	2<br>Favours this option	6<br>Probably favours this option	2<br>Neither favours this option or other options	-<br>Probably favours other options	-<br>Favours other options	**Probably favours this option**
Certainty of the evidence PREFRAIL	1<br>Favours this option	4<br>Probably favours this option	1<br>Neither favours this option or other options	-<br>Probably favours other options	-<br>Favours other options	**Probably favours this option**
Certainty of the evidence FRAIL	-<br>Favours this option	4<br>Probably favours this option	1<br>Neither favours this option or other options	-<br>Probably favours other options	-<br>Favours other options	**Probably favours this option**
Values	-<br>Favours this option	9<br>Probably favours this option	1<br>Neither favours this option or other options	-<br>Probably favours other options	-<br>Favours other options	**Probably favours this option**
Balance of effects	1<br>Favours this option	8<br>Probably favours this option	1<br>Neither favours this option or other options	-<br>Probably favours other options	-<br>Favours other options	**Probably favours this option**

Resources required	1<br>Favours this option	3<br>Probably favours this option	6<br>Neither favours this option or other options	-<br>Probably favours other options	-<br>Favours other options	**Neither favours this option or other options**
Certainty of evidence of required resources	-<br>Favours this option	1<br>Probably favours this option	6<br>Neither favours this option or other options	-<br>Probably favours other options	-<br>Favours other options	**Neither favours this option or other options**
Cost-effectiveness	1<br>Favours this option	1<br>Probably favours this option	5<br>Neither favours this option or other options	-<br>Probably favours other options	-<br>Favours other options	**Neither favours this option or other options**
Equity	3<br>Favours this option	4<br>Probably favours this option	2<br>Neither favours this option or other options	-<br>Probably favours other options	-<br>Favours other options	**Probably favours this option**
Acceptability	1<br>Favours this option	9<br>Probably favours this option	-<br>Neither favours this option or other options	-<br>Probably favours other options	-<br>Favours other options	**Probably favours this option**
Feasibility	-<br>Favours this option	9<br>Probably favours this option	1<br>Neither favours this option or other options	-<br>Probably favours other options	-<br>Favours other options	**Probably favours this option**

- **Nutritional interventions (e.g. diet change, supplementation)**

						DRAFT CONSENSUS JUDGEMNT
Problem	2<br>Favours this option	3<br>Probably favours this option	2<br>Neither favours this option or other options	-<br>Probably favours other options	-<br>Favours other options	**Favours this option**
Desirable effects OVERALL	1<br>Favours this option	4<br>Probably favours this option	4<br>Neither favours this option or other options	-<br>Probably favours other options	-<br>Favours other options	**Probably favours this option (-)**
Desirable effects PREFRAIL	-<br>Favours this option	1<br>Probably favours this option	4<br>Neither favours this option or other options	-<br>Probably favours other options	-<br>Favours other options	**Neither favours this option or other options**
Desirable effects FRAIL	-<br>Favours this option	1<br>Probably favours this option	2<br>Neither favours this option or other options	-<br>Probably favours other options	-<br>Favours other options	**Neither favours this option or other options (+)**
Undesirable effects OVERALL	-<br>Favours this option	3<br>Probably favours this option	6<br>Neither favours this option or other options	-<br>Probably favours other options	-<br>Favours other options	**Neither favours this option or other options**

Undesirable effects PREFRAIL	- Favours this option	- Probably favours this option	5 Neither favours this option or other options	- Probably favours other options	- Favours other options	**Neither favours this option or other options**
Undesirable effects FRAIL	- Favours this option	1 Probably favours this option	3 Neither favours this option or other options	1 Probably favours other options	- Favours other options	**Neither favours this option or other options**
Certainty of the evidence OVERALL	- Favours this option	3 Probably favours this option	6 Neither favours this option or other options	- Probably favours other options	- Favours other options	**Neither favours this option or other options (+)**
Certainty of the evidence PREFRAIL	- Favours this option	2 Probably favours this option	4 Neither favours this option or other options	- Probably favours other options	- Favours other options	**Neither favours this option or other options (+)**
Certainty of the evidence FRAIL	- Favours this option	2 Probably favours this option	3 Neither favours this option or other options	- Probably favours other options	- Favours other options	**Neither favours this option or other options (+)**
Values	1 Favours this option	5 Probably favours this option	2 Neither favours this option or other options	1 Probably favours other options	- Favours other options	**Probably favours this option**

Balance of effects	- Favours this option	7 Probably favours this option	2 Neither favours this option or other options	- Probably favours other options	- Favours other options	**Probably favours this option**
Resources required	1 Favours this option	4 Probably favours this option	4 Neither favours this option or other options	- Probably favours other options	- Favours other options	**Probably favours this option (-)**
Certainty of evidence of required resources	- Favours this option	1 Probably favours this option	5 Neither favours this option or other options	- Probably favours other options	- Favours other options	**Neither favours this option or other options**
Cost-effectiveness	- Favours this option	2 Probably favours this option	4 Neither favours this option or other options	- Probably favours other options	- Favours other options	**Neither favours this option or other options (+)**
Equity	2 Favours this option	5 Probably favours this option	2 Neither favours this option or other options	- Probably favours other options	- Favours other options	**Probably favours this option**
Acceptability	- Favours this option	8 Probably favours this option	- Neither favours this option or other options	1 Probably favours other options	- Favours other options	**Probably favours this option (-)**

Feasibility	- Favours this option	7 Probably favours this option	2 Neither favours this option or other options	- Probably favours other options	- Favours other options	**Probably favours this option (-)**

- **Exercise/physical activity combined with nutritional interventions**

						**DRAFT CONSENSUS JUDGEMNT**
Problem	6 Favours this option	1 Probably favours this option	- Neither favours this option or other options	- Probably favours other options	- Favours other options	**Favours this option**
Desirable effects OVERALL	5 Favours this option	3 Probably favours this option	1 Neither favours this option or other options	- Probably favours other options	- Favours other options	**Favours this option (-)**
Desirable effects PREFRAIL*	2 Favours this option	2 Probably favours this option	- Neither favours this option or other options	- Probably favours other options	- Favours other options	**Favours this option (+)**

Desirable effects FRAIL	2 Favours this option	1 Probably favours this option	1 Neither favours this option or other options	- Probably favours other options	- Favours other options	**Favours this option (-)**
Undesirable effects OVERALL	2 Favours this option	2 Probably favours this option	4 Neither favours this option or other options	1 Probably favours other options	- Favours other options	**Neither favours this option or other options (+)**
Undesirable effects PREFRAIL*	2 Favours this option	1 Probably favours this option	1 Neither favours this option or other options	1 Probably favours other options	- Favours other options	**Probably favours this option**
Undesirable effects FRAIL	1 Favours this option	2 Probably favours this option	1 Neither favours this option or other options	1 Probably favours other options	- Favours other options	**Neither favours this option or other options (+)**
Certainty of the evidence OVERALL	3 Favours this option	3 Probably favours this option	3 Neither favours this option or other options	- Probably favours other options	- Favours other options	**Probably favours this option**
Certainty of the evidence PREFRAIL*	2 Favours this option	3 Probably favours this option	1 Neither favours this option or other options	- Probably favours other options	- Favours other options	**Probably favours this option**
Certainty of the evidence FRAIL	1 Favours this option	3 Probably favours this option	1 Neither favours this option or other options	- Probably favours other options	- Favours other options	**Probably favours this option**

Values	2<br>Favours this option	6<br>Probably favours this option	1<br>Neither favours this option or other options	Probably favours other options	-<br>Favours other options	**Probably favours this option**
Balance of effects OVERALL	2<br>Favours this option	7<br>Probably favours this option	-<br>Neither favours this option or other options	-<br>Probably favours other options	-<br>Favours other options	**Probably favours this option (+)**
Balance of effects PREFRAIL*	2<br>Favours this option	7<br>Probably favours this option	-<br>Neither favours this option or other options	-<br>Probably favours other options	-<br>Favours other options	**Probably favours this option (+)**
Balance of effects FRAIL	2<br>Favours this option	7<br>Probably favours this option	-<br>Neither favours this option or other options	-<br>Probably favours other options	-<br>Favours other options	**Probably favours this option (+)**
Resources required	1<br>Favours this option	3<br>Probably favours this option	6<br>Neither favours this option or other options	-<br>Probably favours other options	-<br>Favours other options	**Neither favours this option or other options (-)**
Certainty of evidence of required resources	-<br>Favours this option	1<br>Probably favours this option	6<br>Neither favours this option or other options	-<br>Probably favours other options	-<br>Favours other options	**Neither favours this option or other options**

Cost-effectiveness	1 Favours this option	1 Probably favours this option	5 Neither favours this option or other options	- Probably favours other options	- Favours other options	**Neither favours this option or other options**
Equity	3 Favours this option	6 Probably favours this option	1 Neither favours this option or other options	- Probably favours other options	- Favours other options	**Probably favours this option (+)**
Acceptability	- Favours this option	10 Probably favours this option	- Neither favours this option or other options	Probably favours other options	- Favours other options	**Probably favours this option**
Feasibility	- Favours this option	8 Probably favours this option	2 Neither favours this option or other options	- Probably favours other options	- Favours other options	**Probably favours this option (-)**

* no studies included in the systematic review on exercise+nutritional interventions on prefrail people

Q2 - Should interventions based on tailored care and/or Geriatric Evaluation and Management (GEM) be recommended to prevent or delay the progression of frailty, or to revert frailty?

(Clinical recommendations - Population perspective - Multiple options)

COMMENTS

None

SUMMARY OF JUDGEMENTS

- **UNI-PROFESSIONAL interventions based on tailored care/GEM**

						**DRAFT CONSENSUS JUDGEMNT**
Problem	- Favours this option	2 Probably favours this option	2 Neither favours this option or other options	1 Probably favours other options	1 Favours other options	**Neither favours this option or other options**
Desirable effects OVERALL	- Favours this option	3 Probably favours this option	6 Neither favours this option or other options	1 Probably favours other options	1 Favours other options	**Neither favours this option or other options**

Desirable effects PREFRAIL	- Favours this option	- Probably favours this option	5 Neither favours this option or other options	2 Probably favours other options	1 Favours other options	**Neither favours this option or other options (-)**
Desirable effects FRAIL	- Favours this option	4 Probably favours this option	2 Neither favours this option or other options	1 Probably favours other options	1 Favours other options	**Probably favours this option (-)**
Undesirable effects OVERALL	1 Favours this option	4 Probably favours this option	4 Neither favours this option or other options	1 Probably favours other options	1 Favours other options	**Neither favours this option or other options (+)**
Undesirable effects PREFRAIL	1 Favours this option	1 Probably favours this option	3 Neither favours this option or other options	2 Probably favours other options	1 Favours other options	**Neither favours this option or other options**
Undesirable effects FRAIL	1 Favours this option	2 Probably favours this option	2 Neither favours this option or other options	2 Probably favours other options	1 Favours other options	**Neither favours this option or other options**
Certainty of the evidence OVERALL	- Favours this option	1 Probably favours this option	7 Neither favours this option or other options	2 Probably favours other options	1 Favours other options	**Neither favours this option or other options (-)**

Certainty of the evidence PREFRAIL	- Favours this option	- Probably favours this option	5 Neither favours this option or other options	1 Probably favours other options	1 Favours other options	**Neither favours this option or other options (-)**
Certainty of the evidence FRAIL	1 Favours this option	- Probably favours this option	5 Neither favours this option or other options	1 Probably favours other options	1 Favours other options	**Neither favours this option or other options**
Values	- Favours this option	6 Probably favours this option	3 Neither favours this option or other options	1 Probably favours other options	1 Favours other options	**Probably favours this option (-)**
Balance of effects\	1 Favours this option	3 Probably favours this option	4 Neither favours this option or other options	2 Probably favours other options	1 Favours other options	**Neither favours this option or other options**
Resources required	1 Favours this option	3 Probably favours this option	5 Neither favours this option or other options	1 Probably favours other options	1 Favours other options	**Neither favours this option or other options**
Certainty of evidence of required resources	1 Favours this option	- Probably favours this option	8 Neither favours this option or other options	1 Probably favours other options	1 Favours other options	**Neither favours this option or other options**

Cost-effectiveness	1<br>Favours this option	2<br>Probably favours this option	7<br>Neither favours this option or other options	1<br>Probably favours other options	-<br>Favours other options	**Neither favours this option or other options**
Equity	-<br>Favours this option	5<br>Probably favours this option	5<br>Neither favours this option or other options	1<br>Probably favours other options	-<br>Favours other options	**Neither favours this option or other options (+)**
Acceptability	2<br>Favours this option	6<br>Probably favours this option	1<br>Neither favours this option or other options	2<br>Probably favours other options	-<br>Favours other options	**Probably favours this option**
Feasibility	2<br>Favours this option	6<br>Probably favours this option	3<br>Neither favours this option or other options	-<br>Probably favours other options	-<br>Favours other options	**Probably favours this option**

- **MULTI-PROFESSIONAL interventions based on tailored care/GEM**

						**DRAFT CONSENSUS JUDGEMNT**
Problem	3<br>Favours this option	2<br>Probably favours this option	1<br>Neither favours this option or other options	-<br>Probably favours other options	-<br>Favours other options	**Probably favours this option**

Desirable effects OVERALL	2 Favours this option	6 Probably favours this option	2 Neither favours this option or other options	1 Probably favours other options	- Favours other options	**Probably favours this option**
Desirable effects PREFRAIL	2 Favours this option	1 Probably favours this option	3 Neither favours this option or other options	2 Probably favours other options	0 Favours other options	**Neither favours this option or other options (+)**
Desirable effects FRAIL	3 Favours this option	3 Probably favours this option	2 Neither favours this option or other options	- Probably favours other options	- Favours other options	**Probably favours this option (+)**
Undesirable effects OVERALL	3 Favours this option	5 Probably favours this option	3 Neither favours this option or other options	- Probably favours other options	- Favours other options	**Probably favours this option**
Undesirable effects PREFRAIL	3 Favours this option	1 Probably favours this option	2 Neither favours this option or other options	2 Probably favours other options	- Favours other options	**Probably favours this option (-)**
Undesirable effects FRAIL	3 Favours this option	1 Probably favours this option	4 Neither favours this option or other options	- Probably favours other options	- Favours other options	**Probably favours this option**

Certainty of the evidence OVERALL	2 Favours this option	2 Probably favours this option	6 Neither favours this option or other options	1 Probably favours other options	- Favours other options	**Neither favours this option or other options (+)**
Certainty of the evidence PREFRAIL	2 Favours this option	1 Probably favours this option	3 Neither favours this option or other options	- Probably favours other options	- Favours other options	**Probably favours this option (-)**
Certainty of the evidence FRAIL	2 Favours this option	2 Probably favours this option	4 Neither favours this option or other options	- Probably favours other options	- Favours other options	**Probably favours this option (-)**
Values	3 Favours this option	5 Probably favours this option	3 Neither favours this option or other options	- Probably favours other options	- Favours other options	**Probably favours this option**
Balance of effects	4 Favours this option	5 Probably favours this option	2 Neither favours this option or other options	- Probably favours other options	- Favours other options	**Probably favours this option (+)**
Resources required	1 Favours this option	7 Probably favours this option	1 Neither favours this option or other options	2 Probably favours other options	- Favours other options	**Probably favours this option**

Certainty of evidence of required resources	1<br>Favours this option	3<br>Probably favours this option	6<br>Neither favours this option or other options	1<br>Probably favours other options	-<br>Favours other options	**Neither favours this option or other options (+)**
Cost-effectiveness	-<br>Favours this option	6<br>Probably favours this option	4<br>Neither favours this option or other options	1<br>Probably favours other options	-<br>Favours other options	**Probably favours this option (-)**
Equity	2<br>Favours this option	6<br>Probably favours this option	2<br>Neither favours this option or other options	1<br>Probably favours other options	-<br>Favours other options	**Probably favours this option**
Acceptability	5<br>Favours this option	6<br>Probably favours this option	-<br>Neither favours this option or other options	-<br>Probably favours other options	-<br>Favours other options	**Probably favours this option (+)**
Feasibility	1<br>Favours this option	7<br>Probably favours this option	2<br>Neither favours this option or other options	1<br>Probably favours other options	-<br>Favours other options	**Probably favours this option**

Q3 - Should "other interventions" be recommended to prevent or delay the progression of frailty, or to revert frailty?

(Clinical recommendations - Population perspective - Multiple options)

COMMENTS

QUESTION:

P2: Psychological interventions were not considered because, I argue, there were no intervention studies (only a cognitive one) about psychological support and coping strategies, as indicated by the qualitative studies. We might indicate that such studies would be needed.

SUMMARY OF JUDGEMENTS

- **Cognitive training (prefrail population)**

						DRAFT CONSENSUS JUDGEMNT
Problem	3<br>Favours this option	2<br>Probably favours this option	1<br>Neither favours this option or other options	-<br>Probably favours other options	-<br>Favours other options	**Probably favours this option (+)**

Desirable effects	2<br>Favours this option	7<br>Probably favours this option	2<br>Neither favours this option or other options	-<br>Probably favours other options	-<br>Favours other options	**Probably favours this option**
Undesirable effects	2<br>Favours this option	3<br>Probably favours this option	6<br>Neither favours this option or other options	-<br>Probably favours other options	-<br>Favours other options	**Neither favours this option or other options (++)**
Certainty of the evidence	Favours this option	6<br>Probably favours this option	4<br>Neither favours this option or other options	-<br>Probably favours other options	-<br>Favours other options	**Probably favours this option (-)**
Values	Favours this option	4<br>Probably favours this option	5<br>Neither favours this option or other options	-<br>Probably favours other options	-<br>Favours other options	**Neither favours this option or other options (++)**
Balance of effects	2<br>Favours this option	6<br>Probably favours this option	2<br>Neither favours this option or other options	-<br>Probably favours other options	-<br>Favours other options	**Probably favours this option**
Resources required	-<br>Favours this option	5<br>Probably favours this option	3<br>Neither favours this option or other options	Probably favours other options	-<br>Favours other options	**Probably favours this option (-)**

Certainty of evidence of required resources	- Favours this option	1 Probably favours this option	8 Neither favours this option or other options	- Probably favours other options	- Favours other options	**Neither favours this option or other options**
Cost-effectiveness	- Favours this option	2 Probably favours this option	7 Neither favours this option or other options	- Probably favours other options	- Favours other options	**Neither favours this option or other options (+)**
Equity	Favours this option	3 Probably favours this option	5 Neither favours this option or other options	Probably favours other options	- Favours other options	**Neither favours this option or other options (+)**
Acceptability	Favours this option	6 Probably favours this option	2 Neither favours this option or other options	Probably favours other options	- Favours other options	**Probably favours this option**
Feasibility	2 Favours this option	7 Probably favours this option	2 Neither favours this option or other options	- Probably favours other options	Favours other options	**Probably favours this option**

- **A composite of exercise + nutritional supplementation + cognitive training (prefrail population)**

						DRAFT CONSENSUS JUDGEMNT
Problem	5<br>Favours this option	1<br>Probably favours this option	-<br>Neither favours this option or other options	-<br>Probably favours other options	-<br>Favours other options	**Favours this option**
Desirable effects	5<br>Favours this option	5<br>Probably favours this option	1<br>Neither favours this option or other options	-<br>Probably favours other options	-<br>Favours other options	**Probably favours this option (++)**
Undesirable effects	2<br>Favours this option	3<br>Probably favours this option	6<br>Neither favours this option or other options	-<br>Probably favours other options	-<br>Favours other options	**Neither favours this option or other options (++)**
Certainty of the evidence	2<br>Favours this option	6<br>Probably favours this option	3<br>Neither favours this option or other options	-<br>Probably favours other options	-<br>Favours other options	**Probably favours this option**
Values	1<br>Favours this option	6<br>Probably favours this option	3<br>Neither favours this option or other options	-<br>Probably favours other options	-<br>Favours other options	**Probably favours this option**

Balance of effects	2<br>Favours this option	5<br>Probably favours this option	2<br>Neither favours this option or other options	1<br>Probably favours other options	-<br>Favours other options	**Probably favours this option**
Resources required	2<br>Favours this option	2<br>Probably favours this option	3<br>Neither favours this option or other options	2<br>Probably favours other options	-<br>Favours other options	**Probably favours this option (-)**
Certainty of evidence of required resources	1<br>Favours this option	1<br>Probably favours this option	7<br>Neither favours this option or other options	-<br>Probably favours other options	-<br>Favours other options	**Neither favours this option or other options (+)**
Cost-effectiveness	2<br>Favours this option	1<br>Probably favours this option	6<br>Neither favours this option or other options	-<br>Probably favours other options	-<br>Favours other options	**Neither favours this option or other options (+)**
Equity	3<br>Favours this option	2<br>Probably favours this option	4<br>Neither favours this option or other options	1<br>Probably favours other options	-<br>Favours other options	**Neither favours this option or other options (+)**
Acceptability	1<br>Favours this option	7<br>Probably favours this option	1<br>Neither favours this option or other options	1<br>Probably favours other options	-<br>Favours other options	**Probably favours this option**

Feasibility	2<br>Favours this option	5<br>Probably favours this option	3<br>Neither favours this option or other options	-<br>Probably favours other options	1<br>Favours other options	**Probably favours this option**

- **Exercise + nutritional consultation (predominantly prefrail population)**

						**DRAFT CONSENSUS JUDGEMNT**
Problem	3<br>Favours this option	1<br>Probably favours this option	2<br>Neither favours this option or other options	-<br>Probably favours other options	-<br>Favours other options	**Probably favours this option**
Desirable effects	2<br>Favours this option	5<br>Probably favours this option	4<br>Neither favours this option or other options	-<br>Probably favours other options	-<br>Favours other options	**Probably favours this option**
Undesirable effects	1<br>Favours this option	3<br>Probably favours this option	6<br>Neither favours this option or other options	1<br>Probably favours other options	-<br>Favours other options	**Neither favours this option or other options (+)**
Certainty of the evidence	2<br>Favours this option	3<br>Probably favours this option	6<br>Neither favours this option or other options	-<br>Probably favours other options	-<br>Favours other options	**Neither favours this option or other options (+)**

Values	- Favours this option	6 Probably favours this option	4 Neither favours this option or other options	- Probably favours other options	- Favours other options	**Probably favours this option (-)**
Balance of effects	2 Favours this option	4 Probably favours this option	4 Neither favours this option or other options	- Probably favours other options	- Favours other options	**Probably favours this option (-)**
Resources required	3 Favours this option	3 Probably favours this option	3 Neither favours this option or other options	- Probably favours other options	- Favours other options	**Probably favours this option**
Certainty of evidence of required resources	- Favours this option	2 Probably favours this option	7 Neither favours this option or other options	- Probably favours other options	- Favours other options	**Neither favours this option or other options (+)**
Cost-effectiveness	1 Favours this option	2 Probably favours this option	6 Neither favours this option or other options	- Probably favours other options	- Favours other options	**Neither favours this option or other options (+)**
Equity	3 Favours this option	3 Probably favours this option	3 Neither favours this option or other options	1 Probably favours other options	- Favours other options	**Probably favours this option**

Acceptability	1<br>Favours this option	8<br>Probably favours this option	1<br>Neither favours this option or other options	-<br>Probably favours other options	-<br>Favours other options	**Probably favours this option**
Feasibility	3<br>Favours this option	7<br>Probably favours this option	1<br>Neither favours this option or other options	-<br>Probably favours other options	-<br>Favours other options	**Probably favours this option**

- **Problem solving therapy (predominantly prefrail population)**

						**DRAFT CONSENSUS JUDGEMNT**
Problem	1<br>Favours this option	1<br>Probably favours this option	1<br>Neither favours this option or other options	1<br>Probably favours other options	2<br>Favours other options	**Neither favours this option or other options (-)**
Desirable effects	-<br>Favours this option	1<br>Probably favours this option	8<br>Neither favours this option or other options	-<br>Probably favours other options	2<br>Favours other options	**Neither favours this option or other options**
Undesirable effects	1<br>Favours this option	-<br>Probably favours this option	10<br>Neither favours this option or other options	-<br>Probably favours other options	-<br>Favours other options	**Neither favours this option or other options**

Certainty of the evidence	- Favours this option	1 Probably favours this option	8 Neither favours this option or other options	- Probably favours other options	2 Favours other options	**Neither favours this option or other options (-)**
Values	- Favours this option	3 Probably favours this option	6 Neither favours this option or other options	- Probably favours other options	1 Favours other options	**Neither favours this option or other options**
Balance of effects	1 Favours this option	2 Probably favours this option	6 Neither favours this option or other options	- Probably favours other options	1 Favours other options	**Neither favours this option or other options**
Resources required	- Favours this option	3 Probably favours this option	5 Neither favours this option or other options	1 Probably favours other options	- Favours other options	**Neither favours this option or other options (+)**
Certainty of evidence of required resources	- Favours this option	- Probably favours this option	9 Neither favours this option or other options	- Probably favours other options	- Favours other options	**Neither favours this option or other options**
Cost-effectiveness	- Favours this option	- Probably favours this option	8 Neither favours this option or other options	- Probably favours other options	1 Favours other options	**Neither favours this option or other options**

Equity	- Favours this option	3 Probably favours this option	5 Neither favours this option or other options	1 Probably favours other options	1 Favours other options	**Neither favours this option or other options**
Acceptability	- Favours this option	7 Probably favours this option	2 Neither favours this option or other options	- Probably favours other options	1 Favours other options	**Probably favours this option (-)**
Feasibility	1 Favours this option	8 Probably favours this option	1 Neither favours this option or other options	1 Probably favours other options	- Favours other options	**Probably favours this option**

- **Hormone therapy (prefrail population – not explicitly defined as such -, males)**

						**DRAFT CONSENSUS JUDGEMNT**
Problem	1 Favours this option	- Probably favours this option	1 Neither favours this option or other options	1 Probably favours other options	3 Favours other options	**Probably favours other options**
Desirable effects	- Favours this option	- Probably favours this option	6 Neither favours this option or other options	2 Probably favours other options	3 Favours other options	**Neither favours this option or other options (--)**

Undesirable effects	1<br>Favours this option	-<br>Probably favours this option	7<br>Neither favours this option or other options	1<br>Probably favours other options	1<br>Favours other options	**Neither favours this option or other options (-)**
Certainty of the evidence	-<br>Favours this option	-<br>Probably favours this option	6<br>Neither favours this option or other options	1<br>Probably favours other options	4<br>Favours other options	**Neither favours this option or other options (--)**
Values	-<br>Favours this option	1<br>Probably favours this option	6<br>Neither favours this option or other options	2<br>Probably favours other options	1<br>Favours other options	**Neither favours this option or other options (-)**
Balance of effects	-<br>Favours this option	-<br>Probably favours this option	5<br>Neither favours this option or other options	2<br>Probably favours other options	3<br>Favours other options	**Neither favours this option or other options (--)**
Resources required	-<br>Favours this option	3<br>Probably favours this option	5<br>Neither favours this option or other options	-<br>Probably favours other options	1<br>Favours other options	**Neither favours this option or other options (+)**
Certainty of evidence of required resources	-<br>Favours this option	2<br>Probably favours this option	7<br>Neither favours this option or other options	-<br>Probably favours other options	-<br>Favours other options	**Neither favours this option or other options (+)**

Cost-effectiveness	-<br>Favours this option	-<br>Probably favours this option	6<br>Neither favours this option or other options	1<br>Probably favours other options	2<br>Favours other options	**Neither favours this option or other options (-)**
Equity	-<br>Favours this option	1<br>Probably favours this option	5<br>Neither favours this option or other options	2<br>Probably favours other options	2<br>Favours other options	**Neither favours this option or other options (-)**
Acceptability	-<br>Favours this option	2<br>Probably favours this option	4<br>Neither favours this option or other options	2<br>Probably favours other options	2<br>Favours other options	**Neither favours this option or other options (-)**
Feasibility	2<br>Favours this option	2<br>Probably favours this option	5<br>Neither favours this option or other options	-<br>Probably favours other options	2<br>Favours other options	**Neither favours this option or other options (+)**
