# Supplementary material for: Interventions to prevent, delay or reverse frailty in older people: a journey towards clinical guidelines
Source: BMC Med. 2019 Oct 29;17:193. doi: 10.1186/s12916-019-1434-2 (PMC6819620; doi:10.1186/s12916-019-1434-2)
Supplement: Supplementary file 5 — Additional file 5. Detailed justification for the judgement upon each Evidence to Decision criterion, for each guideline. [file 12916_2019_1434_MOESM5_ESM.pdf]

FOCUS

FOCUS guidelines: Detailed justification, based on the Evidence To Decision format

GQ - Should interventions to prevent or delay the progression of frailty, or to revert frailty, be adopted in pre-frail or frail older people?

(Clinical recommendation - Population perspective)

DETAILED JUSTIFICATION

- ☒ Problem
- ☒ Desirable effects
- ☒ Undesirable effects
- ☒ Certainty of the evidence
- ☒ Values
- ☒ Balance of effects
- ☐ Resources required
- ☐ Certainty of evidence of required resources
- ☐ Cost-effectiveness
- ☒ Equity
- ☒ Acceptability
- ☒ Feasibility

Problem

The current guidelines work is part of the FOCUS project (664367 — FOCUS — HP-PJ-2014), which was planned as a response to the 3rd Health Programme call (PJ-04-2014) that clearly identified frailty as a priority and asked for the "development of guidelines to address pre-frailty". In response to the call, the FOCUS project was born within the setting of the European Innovation Partnership on Active and Healthy Ageing which was launched by the European Commission as a pilot to tackle the challenge of an ageing population, identifying Frailty as one of the action targets.

Evidence on why frailty should be considered a priority from the population and health systems perspective are comprehensively summarized in the article "Frailty: An Emerging Public Health Priority" (J Am Med Dir Assoc. 2016 Mar 1;17(3):188-92), which was published as a summary of the background document that had been developed by the World Health Organization (WHO) together with the International Association of Gerontology and Geriatrics (IAGG), i.e. the WHO-IAGG Frailty Network, to guide the production of the WHO World Report on Aging and Health and the related Global Strategy and Implementation Plan. Key evidence reported in the paper:

1. The reported prevalence of frailty can vary substantially based on the way frailty was operationalized in the studies. However, considering only studies using the Frailty Phenotype, the data are consistent in providing a prevalence of frailty of around 10% and of pre-frailty of around 40% or more among people aged 65 years and older, with some variability across countries and ethnic groups. It has been well proved that the prevalence increases with older age, and is higher in women and in case of low socioeconomic conditions. The rapidly increase of the mean population age, in high and low income countries, makes the problem urgent.

2. The association between frailty and negative outcomes (including falls, disability, hospitalizations, institutionalization, and mortality) has been repeatedly confirmed regardless of the tools used for its assessment.

3. The frailty condition (especially at its very earliest stages) might be reversible

4. Frailty-related adverse events are patient-centred important outcomes, but are also associated with substantial resource consumption. The identification of successful interventions, whose wide application, even if initially associated with costs of implementation, can finally result into cost savings, represents a priority from the society and health and social systems perspective.

As additional considerations: the implementation of interventions that would delay the progression of frailty makes the identification of people at risk of frail or already frail a priority itself. Considerations concerning level of awareness and attitudes towards the concept of frailty were taken into account for the judgments of the current guidelines. However the current guidelines are not focusing on decisions about specific screening or diagnostic strategies, even if the studies reviewed for the guidelines' purpose might include a screening/diagnostic phase preceding the implementation of the intervention. Frailty may represent an appealing and advantageous approach to age-related conditions, more realistic and appropriate than the one based on "chronological age", and more objective and accurate than the one based on "biological age". However, as the WHO-IAGG Frailty Network document underlines, the care systems, both in high and in middle-low income countries, are either not yet ready for or have so far discouraged a widespread and systematic case finding. An opportunistic detection pattern might be currently the best way to identify people eligible for the interventions whose implementation is here discussed.

Desirable effects, Undesirable effects, Certainty of the evidence

The source of evidence for these criteria was represented by the FOCUS D4.1.2 *Systematic Review: A systematic review of the effectiveness of frailty interventions*. For the FOCUS guideline purposes, the evidence was summarised into 3 evidence tables, one for each specific question, that serve the assessment of each group of interventions (see **Table B1, B2, B3** for details). The panel was also asked for the effort of making an overall judgment on interventions on frailty upon these criteria, despite studies heterogeneity in methods and results. Based on this evidence, the panel was overall in good agreement on judging the anticipated desirable effects of the interventions on frailty taken as a whole as moderate and the quality of evidence as low. There was a lower degree of agreement concerning the anticipated undesirable effects, that were judged as either small/trivial or uncertain; by the way, this heterogeneity was expected given the fact that none of the included studies evaluated outcomes explicitly defined as safety or undesirable.

Values

In the context of the FOCUS project, we did not look for quantitative evidence concerning importance of frailty as an outcome expressed in form of utility values. However we collected qualitative evidence (through focus groups and interviews, and meta-synthesis of qualitative relevant literature) concerning experiences, understanding, and attitudes towards frailty of different stakeholders, i.e. robust, pre-frail and frail older people, caregivers, health and social care professionals, and policy makers [FOCUS Deliverable D4.1 - A synthesis of learning and realities of practice]. The evidence deemed relevant to the judgment upon the level of uncertainty and variability around the importance of frailty as an outcome is here summarised:

• The importance of frailty for older people and caregivers resulted invariably certain. Becoming frail is experienced as a shock by them, or expected with fear, regardless of whether they carry out strategies to prevent or cope with it or not

• Lack of awareness or uncertainty about frailty is still quite common among care providers and policy makers. The existence of different definitions of frailty might enhance this gap. Ageism might also play a role. This might affect how much frailty and interventions on frailty are perceived as important

• Opinions about frailty malleability are different among care providers and policy makers. This increases the uncertainty and variability around the importance of frailty as a target of interventions among these stakeholders

• Uncertainty about ownership of frailty management contributes to generate uncertainty about the importance of frailty among care providers and policy makers.

As additional considerations: from a population perspective, uncertainty and variability of beliefs around the importance of frailty as an outcome, among care providers and policy makers, would not represent a reason to change the strength of recommendations for

interventions on frailty. However this surely plays a role in terms of setting priorities and allocating resources in circumstances of limited availability of human and economic resources.

Balance of effects

Quantitative evidence on the effects of interventions on frailty as a whole and stakeholders' inputs concerning related values (outcome importance) led to make a consensus-based judgment, overall, *probably in favour of their implementation*.

Resources required, Certainty of evidence of required resources, Cost-effectiveness

The FOCUS D4.1.2 Systematic Review looked also for evidence relevant to these criteria. Only 2 studies including health economic analyses on interventions on frailty were retrieved, not performed in Europe, with inconsistent results. An additional evidence source relevant to these criteria would have been represented by the analyses of initiatives (i.e. commitments or Good Practices) within EIP-AHA upon quantitative data upon structure, process and outcome indicators, planned in the FOCUS project – task 5.2. This would have allowed to explore the relationship between the results of interventions in terms of outcomes and the resources implemented. Unfortunately, the paucity of data we were eventually able to collect, despite several attempts and the adoption of different strategies to gather information, prevented us from making any reliable conclusion (D5.2.5). Finally, in FOCUS D5.2.2, 3 and 4 on *Modelling predicted needs and resource consumption*, we demonstrated that physical and cognitive frailty profiles could predict a significant amount of the variance in a range of health and social care use measures and costs. Variables predicted included number of visits to a GP, visits to a specialist/ outpatient appointments, number of nights spent in hospital, visits to or from a nurse, and social care to the home or number of weeks spent in a nursing home. From these results we projected the possible effects of successful interventions on resource consumptions. However, these analyses were not directly answering questions related to the necessary resources to implement physical interventions on frailty, or their cost-effectiveness. Further searches outside the EIP AHA context and on grey literature could have led to more evidence, but they would have gone beyond our project's purposes.

Therefore, for all these reasons, the FOCUS panelists considered the existing evidence upon these criteria, as collected in the frame of the FOCUS project, insufficient to use these criteria to justify their recommendations.

Equity

In terms of the importance of the problem, it is known that the prevalence of frailty is higher in women and in case of low socioeconomic conditions (J Am Med Dir Assoc. 2016 Mar 1;17(3):188-92). This makes the problem more relevant, and the role of interventions on frailty more important, for older people in disadvantaged socioeconomic circumstances.

In terms of evidence regarding differences in the relative effectiveness of interventions, our systematic (FOCUS D4.1.2 Systematic Review: A systematic review of the effectiveness of frailty interventions) and realist (FOCUS D4.1.8 Realist review: A realist review combining findings from the different components to examine what works for whom and in what circumstances) reviews showed that some interventions on frailty (e.g. a nutritional intervention and an intervention based on nurse home visits) resulted more effective in case of study participants with lower socioeconomic status and education. This might be a participant-level effect, i.e. participants in such conditions are more likely to be in a deficit status that makes the intervention necessary and the effect more evident. It can be also a study-level effect since in such studies the comparison group "usual care" corresponded to a lower quality of care, and the intervention differed from it more substantially, compared with studies performed in environments with a usual care that corresponded already to high standards of care.

On the other end, as additional consideration, frailty might limit people mobility and even confine them at home. This might be a limitation for frailer persons to participation in interventions that, although effective, require people to move. Then the adoption of these interventions might discriminate people according to their level of frailty severity and generate

a vicious cycle. Conversely, interventions feasible at or brought to people's home might lead to a fairer distribution of benefits.

Acceptability

Among the stakeholders' inputs collected in the context of FOCUS – WP4, we identified some themes relevant for the assessment of the acceptability of interventions on frailty to stakeholders.

a. The different viewpoints about frailty value and malleability, or the variable level of knowledge about it despite evidence, among *care professionals* and *policy makers* (see *Values* criterion), might affect the perception of the relative importance of interventions on frailty and, hence, their acceptability to those stakeholders, especially if the implementation of such interventions implies the employment of substantial resources and/or additional efforts, and/or requires substantial changes in the system and in the routine practice.

b. The following themes might affect the acceptability of interventions on frailty (in general, or of specific interventions) to *older adults*:

1. The opportunity to intervene on frailty often depends on the identification of people with frailty or at risk of it (i.e. case finding). In particular, in the case of population-based preventive initiatives, interventions might base upon screening programmes. Then, the acceptability of such interventions on frailty might be affected by the acceptability of population-based screening programmes. In fact, qualitative evidence showed that case finding is more acceptable when effective interventions for identified cases are available
2. Differences in cultural preferences concerning aspects that play a central role in some interventions on frailty (like type of patient-care provider relationship, involvement of people's privacy, importance of supervision, etc.) might make acceptability of the same intervention variable across countries, settings and individuals
3. Older adults valued interventions in which participants had choice or autonomy over intervention components, activities and timings
4. Older adults acceptance of interventions on frailty is enhanced in case of interventions that promote their involvement, empowerment, self-advocacy, resilience and include psychological components

Older adults acceptance of interventions on frailty is enhanced in case of interventions that include social connection, rewarding and fun.

Feasibility

One of the main objectives of the FOCUS project has been to identify barriers and facilitators to the implementation and success of interventions on frailty, through the collection of quantitative and qualitative evidence. These evidence have been then critically interpreted according to a realist review approach, with the identification of Contexts, Mechanisms of action and Outcome (CMO) patterns, mapped onto a programme theory model (FOCUS D4.1.8 Realist review: A realist review combining findings from the different components to examine what works for whom and in what circumstances). This led to the identification of facilitators and barriers among participant, organizational, and cultural/geographical factors, which should be taken into account . Thus feasibility of interventions on frailty could not be assessed in absolute terms; the feasibility should be judged contextually, given the opportunity to benefit from facilitators and overcome barriers. However, some considerations from a public health perspective, drawn from the work done in FOCUS-WP4, have a general relevance concerning the current feasibility of such interventions. In the focus groups with older people, caregivers and care professionals, and in the interviews with policy makers, the current inadequacy of care systems was a pervasive theme. There was a general opinion that feasibility of interventions on frailty would come along with the development and adoption of a new management/approach to care, based on a holistic patient-centred approach, on greater integration and coordination across specialties, and on multidisciplinarity. Finances and resourcing also represent barriers to implementation and success, which only an elective rather than compulsory healthcare system could minimise.

The current insufficiency of psychological skills and communication abilities among healthcare professionals surely represents a barrier to implementation and success of interventions on frailty. The need of training in such sense of care professionals has been recognised as a fundamental requirement in most types of interventions, whether based on a physical or on a multidimensional paradigm of frailty.

Adequate training in case of specific interventions has been also recognized as a requirement to overcome specific acceptability and feasibility issues. For instance, adequate training to deliver exercise programmes for pre-frail or frail older people is important to guarantee participants' safety and reduce their reluctance to participate, but also to enhance the perception of safe working conditions of those delivering the interventions.

Q1 - Should physical interventions be recommended to prevent or delay the progression of frailty, or to revert frailty, in pre-frail or frail older people?*

(Clinical recommendation - Population perspective)

** Under the definition of **physical interventions** we included those interventions based on a physical concept of frailty and/or targeting the physical dimension of frailty*

DETAILED JUSTIFICATION

- ☐ Problem
- ☒ Desirable effects
- ☒ Undesirable effects
- ☒ Certainty of the evidence
- ☒ Values
- ☒ Balance of effects
- ☐ Resources required
- ☐ Certainty of evidence of required resources
- ☐ Cost-effectiveness
- ☒ Equity
- ☒ Acceptability
- ☒ Feasibility

Problem

This criterion was used for the assessment of interventions on frailty in general (GQ) and not for the judgment concerning specific types of interventions.

Desirable effects, Undesirable effects, Certainty of the evidence

In the FOCUS D4.1.2 Systematic Review, we included 9 RCTs evaluating the effect of physical interventions on frailty. The results of interventions based on physical activity/exercise programmes were heterogeneous according to the type of programme and frailty definition, and were more favourable when measures of physical frailty were used as outcomes, in the case of interventions delivered in groups and supervised by professionals, regardless of whether mainly prefrail or mainly frail older people participated, rather than in the case of home-based physical activities performed alone. Positive effects, mainly on physical measures of frailty, were found for nutritional interventions based on protein and caloric supplementation, while, in one study, the administration of milk fat globule membrane (MFGM) supplementation was associated with favourable effects only when associated with a group-based exercise program. Overall, the quality of the evidence was low to moderate because of substantial study limitations, inconsistency (different populations and interventions), and imprecision (see table **Table B1** for more details about evidence on effects on frailty as primary outcome). Concerning the effect on secondary outcomes of interest, among the interventions included in the FOCUS D4.1.2 Systematic Review, different types of physical activity programmes (i.e. multicomponent exercise programmes, either individual or group-based, tai chi, and the programme based on Jaques-Dalcroze eurhythmics) were shown to reduce the incidence of and/or injury associated with falls. The effect of physical interventions on function (i.e. the ability to perform activities of daily living, either basic or instrumental) was heterogeneous.

Only one study (Ng et al., Am J Med. 2015;128(11):1225-1236.e1) explored the effect of either an exercise programme alone or in combination with nutritional supplementation on hospitalisation over 12 months, and did not find any statistically significant difference compared with usual care.

Values

In addition to research evidence relative to frailty as an outcome in general (GQ), the following further evidence and considerations were deemed as relevant to recommending physical interventions on frailty or not. Given that frailty can be differently defined, the perceived outcome importance, and importance of interventions that can impact that outcome, might depend on how frailty as an outcome is defined, i.e. what dimensions of frailty are addressed by the intervention. The qualitative evidence we collected in the FOCUS project through focus groups showed that psychological factors of keeping the brain active and engaging socially appeared more important to older adults than their physical ailments – or that their physical ailments were manageable if those other needs were fulfilled. This suggests that frailty is perceived as more important as an outcome when defined, and addressed, according to a holistic paradigm.

Balance of effects

Quantitative evidence on the effects of such types of interventions and stakeholders' inputs concerning related values (outcome importance) led to make a consensus-based judgment, overall, *probably in favour of their implementation*.

Resources required, Certainty of evidence of required resources, Cost-effectiveness

The FOCUS D4.1.2 Systematic Review looked also for evidence relevant to these criteria. However, no studies on economic analyses on interventions on frailty included in Q1 were retrieved. For this reason and for what explained in GQ, the FOCUS panelists considered the existing evidence upon these criteria, as collected in the frame of the FOCUS project, insufficient to use these criteria to justify their recommendations about these interventions.

Equity

In addition to research evidence relative to the equity of interventions on frailty in general (GQ), the following further evidence and considerations were deemed as relevant to recommendations on physical interventions. Our systematic and realist reviews (*FOCUS D4.1.2* and *FOCUS D4.1.8*) showed that some interventions on frailty resulted more effective in case of study participants with lower socioeconomic status and education. In particular this was the case of nutritional interventions based on protein-energy supplementation (Kim & Lee, J Gerontol A Biol Sci Med Sci. 2013;68(3):309-16). It is likely that in such conditions older people are more likely to be in a deficit status, which makes the intervention actually necessary and the effect more evident.

Acceptability

In addition to evidence on acceptability of interventions on frailty in general (GQ), the following qualitative and quantitative evidence, collected within in the FOCUS – WP4, were deemed as relevant to assessing the acceptability of physical interventions:

• Acceptability of exercise programmes to both professionals involved in care provision and older people receiving the intervention, was affected by concerns about safety and adequacy of the programme, especially when self-directed

• Commitment to group-based and expert-led exercise programmes, when delivered at convenient location, was higher and the intervention more successful compared with self-directed individual-based programmes

Feasibility

Some of the research evidence and additional considerations relative to the feasibility of interventions on frailty in general (GQ), are particularly relevant to the implementation of physical interventions on frailty. Among them, some organizational factors that might limit the feasibility of these types of interventions (especially those based on physical exercises) played a particular role in affecting the assessment made by the panel, such as: the need of care professionals with psychological skills and communication abilities that could help motivate and engage people; the need of adequate specific training to deliver exercise programmes for pre-frail or frail older people, in order to guarantee participants' safety and reduce their reluctance to participate, but also to enhance the perception of safe working conditions of those delivering the interventions; the need of facilities to implement group-based supervised interventions (e.g. a gym, dedicated personnel, devices for monitoring) that could favour socialisation and safety.

Q2 - Should interventions based on tailored care and/or Geriatric Evaluation and Management (GEM) be recommended to prevent or delay the progression of frailty, or to revert frailty, in older pre-frail or frail people?*

(Clinical recommendation - Population perspective)

**We included under the definition of "interventions based on tailored care and/or Geriatric Evaluation Management (GEM)" those interventions based on a holistic concept of frailty and on a comprehensive concept of care. Despite sharing this conceptual base, interventions in this group might differ substantially on several aspects, and might be differently classified along those aspects. We framed Q2 upon the distinction between uni-professional and multi-professional interventions, i.e. interventions involving only one type or more types of professionals, respectively. We chose this criterion because it could be more easily related to the required resources. Within these two categories, interventions could be further classified into sub-categories according to:*

- *the care area of professionals primarily delivering the interventions, i.e. whether only health care professionals or not only health care professionals*
- *the setting in which the interventions was delivered, i.e. person's home, primary care, community hospitals, in hospital wards, ED, etc*
- *the type of initiative, i.e. (psycho)educational/consulting interventions or active management (i.e. changing therapy, prescribing visits, planning care pathways, etc)*
- *the type of receiver, i.e. intervention delivered to groups or to individuals*

DETAILED JUSTIFICATION

☐ Problem
☒ Desirable effects
☒ Undesirable effects
☒ Certainty of the evidence
☒ Values
☒ Balance of effects
☐ Resources required
☐ Certainty of evidence of required resources
☐ Cost-effectiveness
☒ Equity
☒ Acceptability
☒ Feasibility

Problem

This criterion was used for the assessment of interventions on frailty in general (GQ) and not for the judgment concerning specific types of interventions.

Desirable effects, Undesirable effects, Certainty of the evidence

In the FOCUS D4.1.2 Systematic Review, we included 10 RCTs evaluating the effect on frailty of different interventions based on a concept of tailored care and/or on GEM. Overall, the results were mixed. The studies were heterogeneous with regards to the characteristics of the intervention and of the participants, and to the type of setting. However, even in the case of similar interventions and settings (i.e. nurse home visits), results were not consistently positive or negative. Multi-professional interventions seemed to work, whether included health and social care professionals or only health care professionals, especially when performed in inpatient settings. Compared with the interventions in Q1, frailty as an outcome was defined more often according to a composite measure taking into account not only the physical domain, but not necessarily using the same definition. Because of such heterogeneity, together with study limitations and imprecision, the overall quality of evidence was judged as low (see table **Table B2** for more details about evidence on effects on frailty as primary outcome). Concerning the effect on secondary outcomes of interest, as expected given the nature of interventions in Q2, studies evaluated those outcomes more likely than in case of studies on physical interventions, but the effects they found were sparse. In one study (Cohen et al., NEJM 2002), neither a GEM-based intervention on inpatients nor a GEM-based intervention on outpatients had a significant effect on mortality, compared with usual care. Conversely, in Monteserin et al. (Fam Pract. 2010), a multi-professional GEM-intervention based on a group session with a nurse, combined with an individual session with a geriatrician for those at risk of frailty, reduced the composite outcome of all causes of death, admissions to nursing home facilities and admissions to a home care programme. Conversely, in Van Hout et al. (J Gerontol A Biol Sci Med Sci. 2010), home visits made by nurses were associated with more accesses to hospital than usual care (likely because of a tighter monitoring and problem finding).

A greater effect on ability to perform activities of daily living, compared with control was found for multi-professional GEM-based interventions, on either inpatients (Cohen et al., NEJM 2002) or patients at discharge form the ED (Eklund et al. BMC Geriatrics 2013). An effect was found also for multi-professional senior group meetings, compared with usual care and with an alternative intervention based on a single uni-professional home visit (Gustafsson et al., J Am Geriatr Soc. 2012), but not in the case of a GEM-based intervention delivered in a community hospital (Li et al., Archives of Gerontology and Geriatrics 2010).

Few studies explored the effect on quality of life. Fairhall et al. (J Am Med Dir Assoc 2015) found no effect of a multi-professional GEM-based programme (EQ-5D). Gustafsson et al. found a significant effect associated both with multi-professional senior group meetings and with the intervention based on a single uni-professional home visit (self-rated health).

In Cohen et al., the GEM-based interventions on inpatients and outpatients was associated with a smaller decrease, on average, of the score on the general health component of the SF-36 tool at discharge (inpatient intervention) and at 12 months (outpatient interventions), compared with usual care. The GEM-based intervention on outpatients was also associated with a higher increase of the score on the mental health component of the SF-36 tool, compared with usual care.

Values

In addition to research evidence relative to frailty as an outcome in general (GQ), the following further evidence and considerations were deemed as relevant to recommending physical interventions on frailty or not. Given that frailty can be differently defined, the perceived outcome importance, and importance of interventions that can impact that outcome, might depend on how frailty as an outcome is defined, i.e. what dimensions of frailty are addressed by the intervention. The qualitative evidence we collected in the FOCUS project through focus groups showed that psychological factors of keeping the brain active and engaging socially appeared more important to older adults than their physical ailments – or that their physical ailments were manageable if those other needs were fulfilled. This suggests that the physical dimension of frailty might be valued less by older adults, and that interventions based only on a physical paradigm of frailty can be perceived as less important (or insufficient) compared with interventions based on a paradigm of frailty that comprises the physical/medical & psychological & social & cognitive dimensions. On the other side, the experiences collected from different stakeholders in the focus groups also suggested that physical frailty might be a trigger for a frailty status that includes other dimensions. For instance, an impaired physical performance (mobility) may reduce one's opportunities for social and psychological engagement, which may deplete mood and affect feelings of well-being. The relationship between the physical and the psychological appeared to be compensatory and synergistic, i.e. that they are bound up together and their co-existence creates an enhanced combined effect. Hence, preventing or delaying physical frailty might be important also for preventing psychological frailty.

Balance of effects

Quantitative evidence on the effects of such types of interventions and stakeholders' inputs concerning related values (outcome importance) led to make a consensus-based judgment, in the overall population, *neither in favour nor against* their implementation, for uni-professional interventions, and *probably in favour of their implementation*for multi-professional interventions.

Resources required, Certainty of evidence of required resources, Cost-effectiveness

The FOCUS D4.1.2 Systematic Review looked also for evidence relevant to these criteria. In the health economic evaluation part of the systematic review, only two studies (Fairhall et al., J Am Med Dir Assoc 2015, and Cohen et al., NEJM 2002) were retrieved. Both dealt with the resources required for the implementation of multi-professional interventions based on tailored care and/or Geriatric Evaluation and Management (GEM) for frailty treatment or prevention.

Fairhall et al. compared usual care with a GEM-based tailored care provided individually to community dwelling frail patients for 12 months by two physiotherapists, a geriatrician, a rehabilitation physician, a dietician, and a nurse. The intervention had an average cost per participant of $1,528.52 (2011 Australian dollars). The calculated incremental cost-effectiveness ratio (ICER) per additional patient experiencing transition from frailty was $15,955. For the "frail" subgroup (3 CHS criteria for frailty) this cost was of $41,428, for the "very frail" subgroup (> 3 CHS criteria) the intervention was dominant (both more effective and less costly than the control). According to a cost-effectiveness acceptability curve, the intervention would be cost-effective with 80% certainty with decision makers' willingness to pay $50,000 per extra person transitioning from frailty.

Cohen et al. randomly assigned hospitalized frail patients, according to a two-by-two factorial design, to receive either care in an inpatient geriatric unit or usual inpatient care, followed (once discharged) by either care at an outpatient geriatric clinic or usual outpatient care. In the geriatric unit and clinic, care was individual-based, and was provided by a geriatrician, a social worker, and a nurse, with availability of preventive and management services (e.g., dietetics, physical and occupational therapy, and clinical pharmacy). Total costs of care (during hospitalization and after discharge, expressed as mean ± SD) were $36,265±1,298 in the inpatient geriatric unit group, $37,292±1,369 in the usual inpatient care group, $35,943±1,292 in the outpatient geriatric clinic group, and $37,608±1,374 in the usual outpatient care group, respectively (US dollars, 1995-1999). Therefor, there was no significant difference in overall costs between experimental and usual inpatient care, or between experimental and usual outpatient care. However, the study failed to show a difference between intervention and control groups in terms of frailty reversal and mortality, even if there was a positive impact on outcomes of functional capacity and mental health.

These findings could help support the feasibility and cost-effectiveness of implementing multi-professional GEM-based interventions, even of theoretically more resource demanding than uni-professional interventions. However, such evidence was judged uncertain by the FOCUS panelists, given the limited number of studies, and their limited generalisability. For this reason, and for what explained in GQ, these criteria were given less weight in the assessment process.

Equity

In addition to research evidence relative to the equity of interventions on frailty in general (GQ), the following further evidence and considerations were deemed as relevant to recommending physical interventions on frailty or not. Our systematic and realist reviews (*FOCUS D4.1.2* and *FOCUS D4.1.8*) showed that some interventions on frailty resulted more effective in case of study participants with lower socioeconomic status and education. In particular this was the case of an intervention based on nurse home visits (Favela et al, Clin Interv Aging. 2013;8:85-95). A possible reason is that in studies conducted in less rich environments the comparison group "usual care" correspond to a lower quality of care, and the intervention differs from it more substantially, compared with studies performed in environments with a usual care that corresponded already to high standards of care.

Acceptability

In addition to evidence on acceptability of interventions on frailty in general (GQ), the following qualitative and quantitative evidence, collected within in the FOCUS – WP4, were deemed as relevant to assessing the acceptability of interventions based on a tailored care concept and/or GEM:

• Given the evidence that acceptance and commitment are increased in case of interventions that put the older person and his/her autonomy of choice at the centre, interventions based on tailored care could be more strongly recommended than fixed, not adaptable, interventions, especially if the older person is effectively involved in making the intervention plan

• However, despite the importance of preserving their autonomy, older people generally showed preferences for face-to face contacts and, in case of psycho-educational interventions, "expert led" sessions rather than self-directed interventions

Feasibility

Some of the research evidence and additional considerations relative to the feasibility of interventions on frailty in general (GQ), are particularly relevant to the implementation of interventions on frailty based on a tailored care concept and/or GEM. In the focus groups with older people, caregivers and care professionals, and in the interviews with policy makers we performed in the context of the FOCUS project, the current inadequacy of care systems was a pervasive theme. There was a general opinion that feasibility of interventions on frailty would come along with the development and adoption of a new management/approach to care, based on a holistic patient-centred approach, on greater integration and coordination across specialties, and on multidisciplinarity. The current insufficiency of psychological skills and communication abilities among healthcare professionals was recognised as another potential barrier to implementation and success of interventions on frailty based on a tailored care approach.

Q3 - *Should other interventions* be recommended to prevent or delay the progression of frailty, or to revert frailty, in older pre-frail or frail people?*

(Clinical recommendation - Population perspective)

**We included in this question those intervention options that do not fit exactly with either (Q1 or Q2) group of interventions. Interventions in this section may differ substantially one from the other. There might be other types of interventions that would match this no-Q1 and no-Q2 definition; currently, we mentioned only those on which we found studies in the systematic review performed in the context of the FOCUS project*

DETAILED JUSTIFICATION

- ☐ Problem
- ☒ Desirable effects
- ☒ Undesirable effects
- ☒ Certainty of the evidence
- ☒ Values
- ☒ Balance of effects
- ☐ Resources required
- ☐ Certainty of evidence of required resources
- ☐ Cost-effectiveness
- ☒ Equity
- ☒ Acceptability
- ☒ Feasibility

Problem

This criterion was used for the assessment of interventions on frailty in general (GQ) and not for the judgment concerning specific types of interventions.

Desirable effects, Undesirable effects, Certainty of the evidence

In the FOCUS D4.1.2 Systematic Review, there were 3 RCTs evaluating the effect on frailty of the 5 different intervention options here assessed, compared with usual care. Evidence for each option came only from one study. Hence, neither analytical nor descriptive synthesis was possible. Briefly, in the same study of overall good quality exploring also other options (Ng et al., Am J Med 2015), cognitive training, was shown to passively affect the natural history of frailty, even promoting a transition to a lower Fried's category, with a greater effect when combined with exercise and nutritional supplementation (also compared with the intervention based only on exercise or only on nutritional supplementation). In the same study, No effect on frailty defined according to physical strength indicators was seen for 3 different hormone therapies, i.e. dehydroepiandrosterone (DHEA) and/or atamestane (Muller et al., J Clin Endocrinol Metab 2006). The intervention based on exercise plus diet consultation during the exercise was included in Q3 even if representing in part a physical intervention, given the particular nature of the nutritional intervention and the way it was combined with the exercise. It was shown to be associated with a greater improvement in frailty score, compared with no intervention, at the end of the intervention (3 months) but not at 6 or 12 months. The problem solving therapy intervention evaluated in the same study (Chan et al. BMC Geriatrics 2012) was shown to have no effect on frailty score at any time. All these studies included pre-frail populations, differently defined (see **Table B3** for more details about evidence on effects on frailty as primary outcome). Concerning the effect on secondary outcomes of interest, in Chan et al., neither the exercise + nutritional consultation intervention nor the problem solving therapy was associated with a significant effect on cognitive and mental performance (Mini Mental State Examination, Primary Care Evaluation of Mental Disorders [PRIME-MD], function (Barthel Index), and quality of life (EQ-5D). In Ng et al. neither the cognitive training alone nor its combination with the exercise programme and nutritional supplement had a significant effect on function, falls, and hospitalization. The certainty of evidence was judged overall low since the evidence comes from only one study per intervention, and the studies were quite small and with some methodological pitfalls.

Values

No specific evidence on values (outcome importance) was considered for the assessment of the interventions in Q3. However, some of the evidence concerning the importance of frailty according a mainly physical paradigm (see Q1), and according a more holistic concept (see Q2) was also relevant to the assessment of some of the interventions in Q3, according to the nature of the intervention assessed.

Balance of effects

Quantitative evidence on the effects of such types of interventions and stakeholders' inputs concerning related values (outcome importance) led to make a consensus-based judgment,

probably in favour the implementation of interventions based on cognitive training alone or combined with exercise and nutritional supplementation; *probably in favour*, but with more heterogeneity across the panelists, for exercise combined with diet consultation; and *neither in favour nor against* the problem-solving therapy option (with some voting *in favour*) and the hormone therapy (some voting *against*).

Resources required, Certainty of evidence of required resources, Cost-effectiveness

The FOCUS D4.1.2 Systematic Review looked also for evidence relevant to these criteria. However, no studies on economic analyses on interventions on frailty included in Q3 were retrieved. For this reason and for what explained in GQ, the FOCUS panelists considered the existing evidence upon these criteria, as collected in the frame of the FOCUS project, insufficient to use these criteria to justify their recommendations about these interventions.

Equity

The evidence and additional considerations on general aspects related to the equity criterion (GQ) were taken into account. In addition, the fact that the hormone therapy intervention was directed only to males was deemed as relevant to the assessment upon equity.

Acceptability

Some of the evidence and additional considerations specific either for physical interventions (Q1), or for interventions on patient-centred approach and comprehensive care (Q2), were relevant also to some of the interventions in Q3, according to the nature of the intervention assessed.

Feasibility

Some of the evidence and additional considerations specific either for physical interventions (Q1), or for interventions on patient-centred approach and comprehensive care (Q2), were relevant also to some of the interventions in Q3, according to the nature of the intervention assessed.
